# Analysis of Pesticide Levels in Honey and Pollen from Irish Honey Bee Colonies Using a Modified Dutch Mini-Luke Method with Gas and Liquid Chromatography–Tandem Mass Spectrometry Detection

**DOI:** 10.1021/acs.jafc.3c02250

**Published:** 2023-08-16

**Authors:** Marcela A. Díaz, Darren P. O’Connell, Seana Jordan, Catriona O’Connor, Paul Martin, Julia C. Jones, Jim Garvey

**Affiliations:** † School of Biology and Environmental Science, University College Dublin, Belfield, Dublin D04 N2E5, Ireland; ‡ Food Chemistry Division, Department of Agriculture, Food and The Marine, Celbridge W23 X3PH, Ireland

**Keywords:** pesticide detection, Dutch mini-Luke extraction, honey bee, honey, pollen, GC-MS/MS, UHPLC-MS

## Abstract

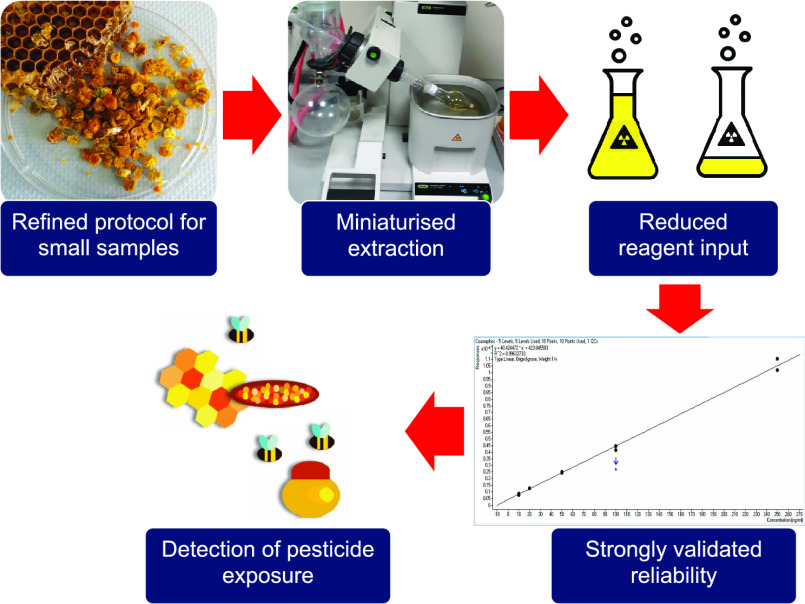

Determining the levels of agrochemicals, such as pesticides, that honey bees are exposed to is critical for understanding what stress factors may be contributing to colony declines. Although several pesticide detection methods are available for honey, limited work has been conducted to adapt these methods for pollen. Here, we address this gap by modifying the Dutch mini-Luke extraction method (NL method) for pesticide analysis in honey and pollen from throughout the island of Ireland. The NL method was modified to enable detection in small-sized samples and validated for both pollen and honey matrices. The modified NL method combined with liquid and gas chromatography–tandem mass spectrometry gave consistent results in terms of accuracy and precision measured by recovery experiments and was successfully applied in the analysis of a range of pesticide residues. The modified NL method developed here provides a key tool for detecting pesticides in honey bee colony resources and the environment more broadly.

## Introduction

In recent decades, declines in honey bee (*Apis mellifera*) colony numbers have been reported with pesticides being identified as one of the contributing stressors.^1^ It has been suggested that pesticides play a role in colony collapse disorder (CCD), a phenomenon characterized by a high rate of honey bee colony losses.^2,3^ Synthetic agrochemicals are particularly concerning, including systemic and persistent substances like neonicotinoids, which can accumulate in pollen and nectar of treated and untreated flowers, as well as in soil and waterways.^4−6^ When foraging worker bees collect these polluted resources, agrochemicals can be transported back to the colony and contaminate beeswax and other adult bees, developing brood and potentially contributing to colony collapse.^4,7−9^

Accurate detection of pesticides in honey bee colony resources is crucial for assessing the levels of pesticides bees are exposed to within the hive and in the wider environment.^10^ Moreover, it helps to determine the safety of these resources for human consumption and to assess the impact on other insects that are utilizing similar resources.^10,11^ Maximum residue levels (MRLs) have been set by the European Union (EU) to regulate the appropriate use of pesticide products and are key considerations when conducting chemical analyses.^11^

There are three main extraction methods commonly used for the multiresidue analysis of pesticides in food matrices such as honey: QuEChERS, the Swedish ethyl acetate (SweEt) method, and the NL method.^10,12−16^ While the QuEChERS method is widely used due to its rapid implementation and its minimal equipment requirements, it often produces quite dirty extracts with significant quantities of matrix co-extracts and may result in low recoveries of pH-sensitive pesticides.^17^ Similarly, the SweEt method, commonly used for the analysis of fruits and vegetables, requires additional cleaning steps to remove matrix contaminants which may affect the recovery of some compounds.^15^

The NL method is a good all-round extraction method that works well with various pesticide–matrix combinations including fruits, vegetables, honey, and cereals.^13,18,19^ It provides clean extracts and gives better recoveries with pH-sensitive compounds.^20^ Moreover, it allows for the detection of multiple classes of compounds such as organochlorines, organophosphates, organonitrogen, hydrocarbons, and neonicotinoids, and it is suitable for the recovery of polar and nonpolar compounds.^13,16^ Further, the NL method is compatible with numerous multiresidue detection technologies such as gas chromatography–tandem mass spectrometry (GC-MS/MS), liquid chromatography–tandem mass spectrometry (LC-MS/MS), high-performance liquid chromatography (HPLC), etc.^21,22^ The Luke method, known for its reliability in detecting pesticides in fruits and vegetables since the 1980s,^23^ has been used in the Food and Drug Administration (FDA) pesticide residue analysis and remains the preferred choice for residue analysis in federal and state laboratories in the United States,^19,20^ as well as in some European countries.

Recent studies have addressed the environmental impact associated with the utilization of large volumes of solvents in the NL method.^16,24^ For instance, it has been demonstrated that minimizing the volume of solvents used during extraction reduces the environmental footprint, while maintaining accuracy in pesticide detection.^16,24^ However, despite the risks associated with exposure to pesticide-contaminated pollen in several key beekeeping regions worldwide, little has been done to adapt the extraction methods for the analysis of the pollen matrix.^25−27^ The limited sample volumes of pollen samples, typically ranging from 0.1 to 5 g, further compounds the issue.

To address these gaps, we have modified the NL method to facilitate the detection of pesticides in small sample volumes of the key honey bee colony resources, pollen and honey, collected throughout the island of Ireland. Further, we have used this modified method to investigate the levels of 346 pesticides, active substances, and metabolites, which are part of the analytical scope of the Irish Department of Agriculture, Food and Marine. Notably, to the best of our knowledge, this is the first time the NL method has been adapted and applied for detecting pesticides in honey and pollen samples.

## Materials and Methods

### Chemicals and Reagents

To avoid contaminants and reduce background noise during extraction and detection,^28^ all solvents (acetone, petroleum ether [40–60 °C], dichloromethane, methanol, and ethyl acetate) were of pesticide grade, and anhydrous sodium sulfate was of analar (high purity) grade. The anhydrous sodium sulfate was heated at 300 °C for 4 hours and cooled down to room temperature before extraction to ensure complete dehydration. Honeywell Research Chemicals and Fisher Scientific provided all of the chemicals as well as some materials (e.g., 250 mL poly(tetrafluoroethylene) [PTFE] centrifuge tubes, 250 mL rounded-bottom flasks, and 0.2 μm syringe sterilized disposable filters). All analytical standards were provided by LGC U.K., Sigma-Aldrich (Ireland), and Analab Ireland Ltd.

To enable the detection of 346 pesticides, the NL method was integrated with gas chromatography–tandem mass chromatography (GC-MS/MS) and ultrahigh-performance liquid chromatography integrated with mass spectrometry (UHPLC-MS), which allow the detection of 180 and 166 compounds, respectively. Pure individual standards for all of the analytes in the scope of this analysis were made up in either acetone/hexane (90:10) or ethyl acetate at a concentration of 300–600 mg/L. In addition, spiking standards or pesticide standards were prepared as follows: One GC-spike for GC-MS/MS in ethyl acetate and two LC-spikes for UHPLC-MS prepared in methanol consisting of 128 analytes for detection through electrospray ionization (ESI) in positive mode (ESI^+^ spike) and 38 pesticides for pesticide detection in negative mode (ESI^–^ spike). All spiking standards were prepared at a final concentration of approximately 1 mg/L and used for recovery studies. In addition, calibration standards were prepared for GC-MS/MS from the spiking standards at twice the required concentration to allow for matrix matching. The calibration standards were matrix-matched with the sample extract, honey or pollen as appropriate, to minimize matrix effects and improve quantification. Calibration standards were used to construct a calibration curve which served to determine linearity. Calibration standards were not prepared for UHPLC-MS. Instead, solvent standards were used for calibration by UHPLC-MS. Also, to mitigate the matrix effect in UHPLC-MS, the sample extracts were diluted in methanol at a ratio of 1/20.

### Sample Preparation

For recovery experiments, samples of honey and pollen collected by beekeepers across Ireland (refer to the Analysis of Real Samples section) larger than 30 g were tested for pesticides using the Dutch mini-Luke extraction method described below and samples reported as clear (no pesticides greater the lower calibration level) were used as blank samples of each matrix. These blank samples were fortified with the spiking solutions pre-extraction, and the calculated recovery was used for quality control of the extraction process and to validate the method. In addition, one honey sample (ID: 804-194) provided by the Pesticide Residues Laboratory from the Department of Agriculture, Food and Marine, which was positive for boscalid, was used as a quantification check of the modified extraction method.

### Dutch Mini-Luke Extraction Method

The extraction method used in this study is a modified version of the Dutch mini-Luke extraction method (NL method). 15 g of sample, either honey or pollen, is combined with 10 mL of water in a 250 mL PTFE centrifuge tube. Then, the mixture is extracted with acetone (30 mL) and homogenized using a T25 digital Ultra-Turrax blender (IKA, Germany). Following this, 30 mL each of petroleum ether and dichloromethane is added to the mixture. To remove residual water,^16,20^ 30 g of anhydrous sodium sulfate is added into the sample and the sample is homogenized again. The resulting mixture is centrifuged at 3500 rpm for 5 min. Next, 2/3 of this extract (60 mL) is taken and transferred to a 250 mL round flask. The extract is concentrated to a low volume (∼2 mL) using a BÜCHI rotavapor spinning at 200 rpm at 40 °C. Subsequently, 15 ml of ethyl acetate is added and the extract is once again reduced to a low volume (∼2 mL). The concentrated extract is then transferred to a 10 mL volumetric flask, and any remaining sample remnants are rinsed with ethyl acetate. The residual solvent is added to the volumetric flask, and the volume is adjusted to 10 ml using ethyl acetate. If the gas chromatography (GC) fraction appears cloudy due to the presence of solid residues, the aliquot is transferred to a 50 mL conical centrifuge plastic tube to be cooled down to −20 °C for 15 min and subsequently centrifuged at 4000 rpm for 3 min. Finally, prior to analysis, all sample extracts are filtered using a disposable plastic syringe fitted with a 0.2 μm filter. The ethyl acetate extract is then directly analyzed using GC-MS/MS, and for UHPLC-MS analysis, the ethyl acetate extract is diluted with methanol (1:20).

A honey or pollen blank, with double the amount of sample (30 g), is also included in the extraction process to provide blank matrix for matrix matching. In addition, two separate blank samples are spiked and introduced into the extraction process as a quality control check. The first blank is spiked with 1.5 mL of the GC spiking solution for a target recovery of 100 μg/kg. The second blank is spiked with 0.75 mL of each liquid chromatography (LC) spiking mixture (ESI^+^ spike and ESI^–^ spike) for a target recovery of 50 μg/kg. The NL method yielded a sample with a matrix concentration of 1 g/mL.

### Modification of the Extraction Method

Because pollen samples tend to be small, we miniaturized the extraction method described above and adapted it for 5 g of pollen or honey. To achieve this, one-third of solvents (10 mL) were utilized and the solvent exchange was performed by collecting the full extract (supernatant), approx. ∼25 mL. Next, solvent reduction was done as in the NL method previously described. After the second reduction, the extract was transferred to a 5 mL volumetric flask and made up to volume with ethyl acetate, resulting in a concentration of 0.83 g/mL. An aliquot of this extract was used for the GC-MS/MS analysis. Clean-up steps were carried out as required as described above. Finally, to obtain the LC fraction, 0.5 mL of the GC fraction was diluted in methanol in a 10 mL volumetric flask and the aliquot resulted in a 1–20 dilution.

### Chemical Analysis

Pesticide residue analysis was performed by gas chromatography–tandem mass chromatography (GC-MS/MS) and ultrahigh-performance liquid chromatography (UHPLC-MS). In some cases, where the MS/MS data was ambiguous due to poor linearity or low recovery, the extracts were analyzed by GC–high-resolution accurate mass screening to confirm the results.

### Gas Chromatography–Tandem Mass Spectrometry (GC-MS/MS)

The GC-MS/MS analysis was performed using an Agilent 7890 instrument equipped with an electron ionization (EI) ion source, with a fixed voltage of 70 eV. This instrument was connected to an Agilent 7000 triple quadrupole mass spectrometer. The mass spectrometry parameters were set as follows (Table S1): ion source temperature, 280 °C; MS1 and MS2 temperature, 150 °C; ionization mode as EI; and data profile. The GC-MS detector operates in pulsed splitless injection mode at 124 psi and utilizes a fused silica capillary Agilent J&W HP5 MS GC column (15 m × 0.250 mm × 0.25 μm). The injection was performed at 80 °C (Table S1), and helium (2.25 mL/min) was used as a carrier. The retention time was locked using the analyte ppDDE at 24.02 ± 0.01 min.

### Ultrahigh-Performance Liquid Chromatography–Tandem Mass Spectrometry (UHPLC-MS)

UHPLC used was the Agilent 6490, connected to an Agilent 7000 triple quadrupole mass spectrometer. The analytical column was a Phenomenex Kinetex C18 core–shell column (150 mm × 4.6 mm). The mobile phase consisted of ammonium phosphate (5 mmol) in deionized water (A) and methanol (B). Pesticides were separated using a solvent gradient constructed from Buffer A (99% water) and Buffer B (99% methanol) (Table S2). The flow rate was 0.5 mL/min, and the analysis time was 35 min. For mass spectrometry, an ESI source was used for multi-reaction monitoring, operating in positive and negative modes. Detailed UHPLC-MS parameters can be found in Table S3. For both GC-MS/MS and UHPLC-MS, the software used for the analysis was Agilent Mass Hunter.

### Identification and Quantification of Pesticides

**Figure 1:**
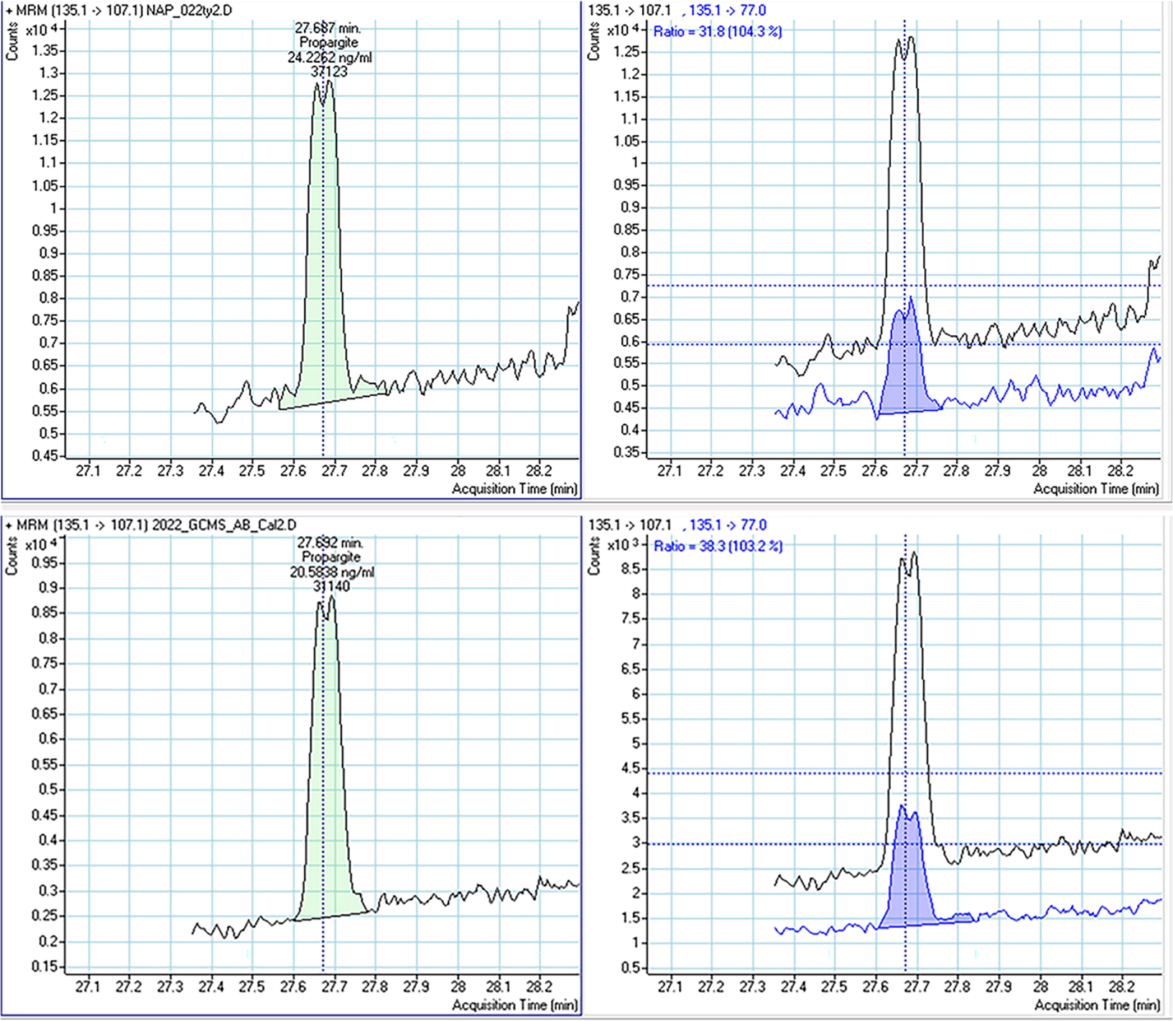
Chromatogram of propargite detected in honey. The chromatogram exhibits two pairs of transitions (T), T1 (left):135.1 → 107.1 *m*/*z* and T2 (right): 135.1 → 77.0 *m*/*z*. The retention time for propargite was set at 27.692 min. The ratio of the two transitions observed in the sample (top pair) falls within the acceptable tolerance range, and both, the parent and product ion peak shape, resemble those presented by the calibration standard (bottom pair) at the second level (20 μg/kg). This confirms the presence of the compound propargite in the sample.

For each compound in the quantification and screening method, two transitions were generated. One transition was used for the identification and quantification of the pesticide, while the second transition was used for confirming the identity (Figure 1). To determine positive results, the transitions observed in the sample were compared to those of the corresponding calibration standard level (Figure 1). The transitions for all of the compounds used in this study had been optimized specifically for the detection of pesticides in fruit and vegetables (Table S4). Further, samples with pesticide concentration higher than the limit of quantification (LOQ), the lowest concentration determined by validation experiments (see below), were reported as positives.

### Validation of the Modified Method

The miniaturized method was validated following the guidelines outlined in the SANTE document (SANTE/11312/2021), which is available on the website of the EU Reference Laboratory for Pesticides (www.eurl-pesticides.eu).^29^ The validation of the method included the assessment of different parameters, including linearity, recovery, repeatability, and matrix effect. Linearity and recovery were measured for all of the experiments and 346 compounds consisting of 180 for GC-MS/MS and 166 for UHPLC-MS.

#### Linearity

To measure linearity, two sets of bracketing calibration standards were used to generate a curve consisting of five calibration levels with duplicate points at each level (Figure S1). The calibration levels for the GC-MS/MS analysis were 10, 20, 50, 100, and 250 μg/kg using matrix matched standards. For the UHPLC-MS analysis, the calibration levels were 0.5, 1.0, 5.0, 10, and 12.5 μg/kg, with a dilution factor of 20 giving an equivalent concentration range of 10–250 μg/kg in the samples. The calibration curves were not forced through the origin. The coefficient of determination (*R*^2^) was used to evaluate linearity. Values above 0.950 were considered as acceptable.^29^

#### Recovery and Repeatability

Recovery experiments were conducted by fortifying seven honey blank samples at 10 μg/kg and six honey blanks at 100 μg/kg with standard mixtures. According to the guidelines from the SANTE/11312/2021, the acceptable average recovery falls in the range of 70–120%,^16,29^ which gives an estimate of trueness. A range of 60–140% is also considered practical in routine analysis for individual pesticides.^20,29^ In this experiment, average recoveries outside the range of 60–140% were considered unacceptable. Recovery was calculated using the following formula (eq 1^30^).
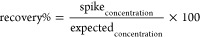
1The replicates were used to calculate the mean recovery, standard deviation, and relative standard deviation (RSD) for all of the analytes tested in each validation batch. The mean recovery provides an estimate of the method’s trueness, while the RSD gives an estimate of the precision.^30^ For repeatability experiments, an RSD within ± 20% was considered acceptable.^16,20,29^

#### Matrix Effect

The analysis of the matrix effect plays a key role in enhancing the accuracy of the method by identifying bias in recovery calculation.^31^ This allows the incorporation of steps in the method to mitigate any matrix effect while measuring the target compounds. In this study, the matrix effect was assessed in honey and pollen using GC-MS/MS. The matrix effect was not considered for liquid chromatography as UHPLC-MS aliquots were diluted (1:20) to minimize the effect. Here, to assess the matrix effect, five samples of each matrix, previously analyzed and confirmed to be free from residues, were combined (matrix-matched) with the calibration standards at five different levels: 10, 20, 50, 100, and 250 μg/kg and a calibration curve was constructed from these matrix-matched standards. The run was bracketed by two sets of calibration standards prepared in solvent (ethyl acetate). To determine the matrix effect, the slope of the calibration curve generated from the solvent standards was compared with the slope of the calibration curve constructed from the matrix-matched standards.^32^ The difference between the slopes determines the matrix effect, which can be a signal increase or decrease.^32^ The result was expressed as a percentage of matrix effect, as shown in eq 2.^20,33^ According to the SANTE guidance document (SANTE/11312/2021), a percentage of matrix effect outside the range of −20 to 20% is considered significant.^29^
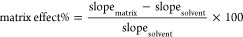
2

### Analysis of Real Samples

Once validated, the modified method was used to analyze 99 pollen samples and 92 honey samples collected by beekeepers as part of Ireland’s National Apiculture Program (NAP). The NAP is a research program funded by the European Union and the Department of Agriculture, Food and Marine, Ireland, which aims to investigate honey bee health in Ireland. Beekeepers from throughout the island of Ireland collected pollen and honey from their colonies using standardized sampling protocols outlined by the NAP. Samples were collected in 2020 and 2021. Briefly, each beekeeper sampled up to five of their hives, and those with fewer colonies sampled all of them. For pollen collection, a section of comb (approx. 10 cm × 10 cm) containing stored pollen was cut from a frame from each colony and stored in a designated cardboard box. In addition, honey was collected directly from the comb in each colony and stored in a designated tube (50 ml centrifuge conical plastic tube). All pollen and honey samples were stored at −20 °C immediately after collection. Then, the samples were transported on ice to the School of Biology and Environmental Sciences at University College Dublin. All samples were then stored at −80 °C for analysis.

To enable solvent extraction for chromatographic analysis, stored pollen was removed from the comb using a stainless steel long-handle micro lab scoop, avoiding wax particles. This analysis prioritized fresh stored pollen which can be identified by its vibrant color and dried, fine-powdery, compact appearance. Beebread, on the other hand, which has been characterized as fermented pollen mixed with nectar,^34,35^ was included only when an insufficient amount of fresh stored pollen was available. Samples collected by each beekeeper (from each apiary) were pooled and homogenized. Pollen and honey samples were weighed at room temperature and stored at −20 °C. Then, the samples were removed from the fridge 2 h before solvent extraction.

## Results

### Validation of the Miniaturized Method

The NL method, originally designed for extracting pesticides from 15 g of sample, was modified to facilitate the extraction of pesticides from 5 g of honey and pollen. The validation of the modified method was conducted following the European guidelines (refer to methodology, Section 2.5).

In this study, the limit of quantification (LOQ) was set at the lower calibration level for which good precision and accuracy were achieved without exceeding the default maximum residue levels (MRLs). LOQ was determined as 10 μg/kg for 318 out of 346 compounds (Table S2).

Overall, the modified NL method demonstrated excellent performance in extracting targeted analytes from honey samples. Specifically, over 84.4% (152 out of 180) of the analytes met the criteria for recovery, repeatability, and linearity when analyzed using GC-MS/MS at 100 μg/kg. Similarly, with UHPLC-MS analysis, approximately 83.1% (138 out of 166) of the targeted analytes exhibited satisfactory results under the same parameters, measured at a spiking level of 100 μg/kg, while at a lower concentration of 10 μg/kg, 77.8% of the compounds were successfully validated using GC-MS/MS and 76.5% were validated using UHPLC-MS. This highlights the robustness and reliability of the modified NL method as well as its effectiveness in detecting and quantifying targeted analytes.

#### Linearity Data

The correlation coefficient (*R*^2^) was used to evaluate linearity. *R*^2^ values above 0.950 were considered acceptable. Linearity experiments were performed in honey samples. The results indicate that linearity was demonstrated by an average of 89.0% of the analytes evaluated through GC-MS/MS and UHPLC-MS. Among the validated compounds (308 out of 346 compounds), the *R*^2^ values ranged from 0.956 to 0.999 in GC-MS/MS experiments and from 0.950 to 0.998 in UHPLC-MS experiments, illustrating the reliability of the miniaturized method (Table S5). Thirty-eight out of 346 pesticides analyzed by liquid and gas chromatography gave poor linearity, with a coefficient of determination ranging from 0.176 to 0.949 (Table 1).

**Table 1:** Pesticides That Did Not Meet the Validation Criteria

GC compounds		LC compounds	
azinphos-methyl	fenvalerate-II	BAC14	thiophanate-methyl
azoxystrobin	tau-fluvalinate-I/-II	BAC16	2,4,5-T
binapacryl	folpet	cyproconazole II	bixafen
captafol	HCH-α	difenoconazole	chlorfluazuron
captan	heptachlor	dodine	cycloxydim
cyfluthrin	methoxychlor	methiocarb Sulfone	diflubenzuron
cypermethrin	permethrin-I	spirotetramat	fluazifop
dicofol	phorate	thiabendazole	flubendiamide
etoxazole	ppDDT	thiophanate-ethyl	haloxyfop
etridazole	propargite		

#### Recovery and Repeatability Data

Repeatability experiments were carried out on honey samples at two spiking levels to evaluate trueness and precision using both GC-MS/MS and UHPLC-MS (Table S5). For acceptable results, the average recovery was expected to fall within the range of 60–140% with an RSD% below 20% (SANTE/11312/2021). In this study, 84.7% of the compounds analyzed (346) at a spiking level of 10 μg/kg and 89.9% of the pesticides studied at a spiking level of 100 μg/kg were successfully validated. However, 35 (10.1%) out of the total 346 compounds were not validated, as they did not meet the validation criteria for repeatability based on the recovery data (Table S5).

**Figure 2:**
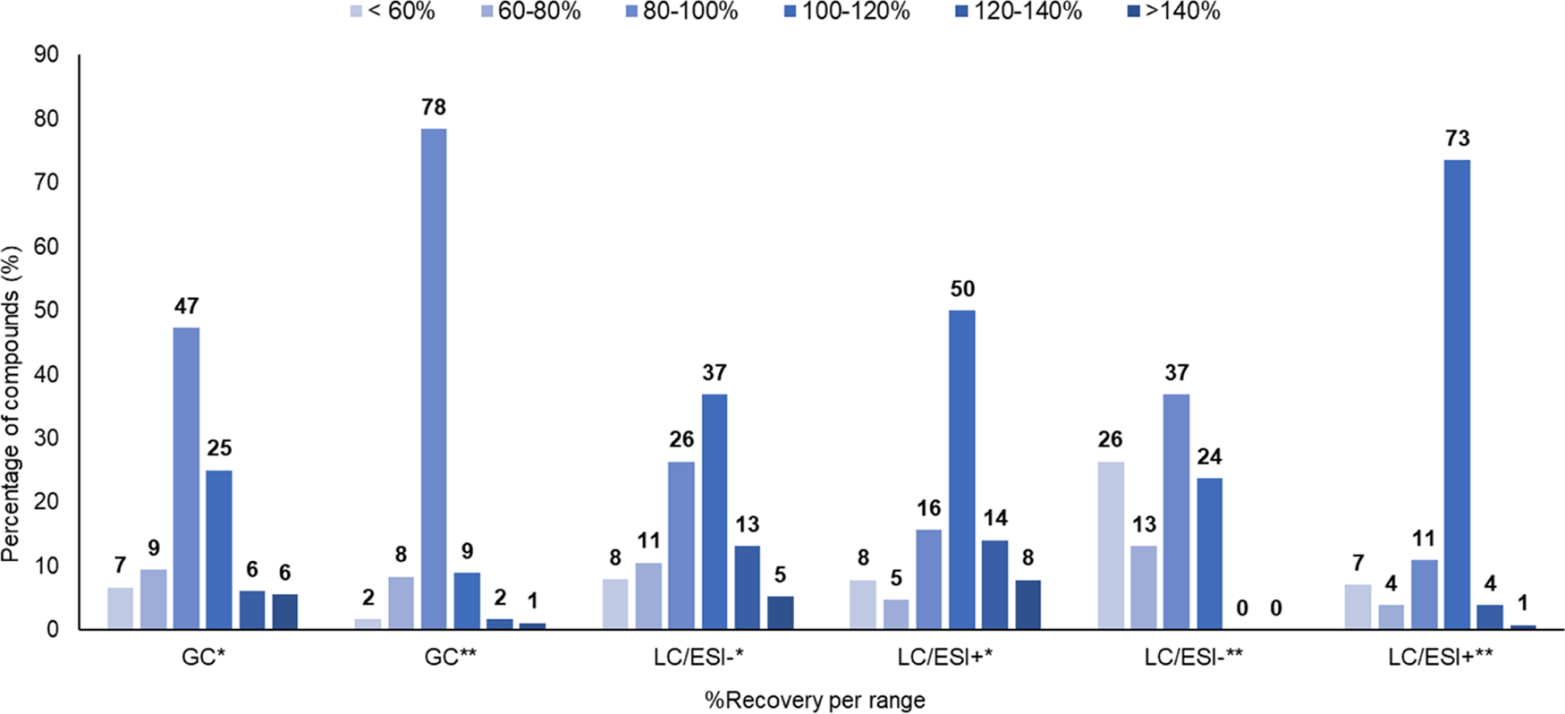
Recovery data for 180 pesticides analyzed by GC-MS/MS and 166 compounds analyzed by UHPLC-MS. Two different concentration levels were evaluated: 10 μg/kg (*) and 100 μg/kg (**). The abbreviations GC and LC represent GC-MS/MS and UHPLC-MS, respectively. The ESI operating mode is shown as ESI^+^ for positive mode, and ESI^–^ for negative mode. The data is presented as a percentage of compounds per percentage of recovery, categorized into six ranges from <60 to >140%.

In GC-MS/MS, out of 180 compounds, a total of 147 (81.7%) met the validation criteria at the 10 μg/kg spiking level, while 170 (94.4%) compounds met the validation criteria at 100 μg/kg (Figure 2). Similarly, in UHPLC-MS, 166 pesticides were evaluated, resulting in the validation of 136 pesticides (81.9%) at 10 μg/kg and 140 pesticides (84.3%) at 100 μg/kg (Figure 2). Notably, nine compounds (binapacryl, captafol, captan, cypermethrin, dicofol, etoxazole, fenvalerate-II, folpet, and spirodiclofen) were not detected by GC-MS/MS at 10 μg/kg. Likewise, methomyl was not detected at 10 μg/kg and MCPA was not detected at 100 μg/kg by the UHPLC-MS. Moreover, dodine and cycloxydim were not detected by the UHPLC-MS at any concentration level.

#### Matrix Effect Data

**Figure 3:**
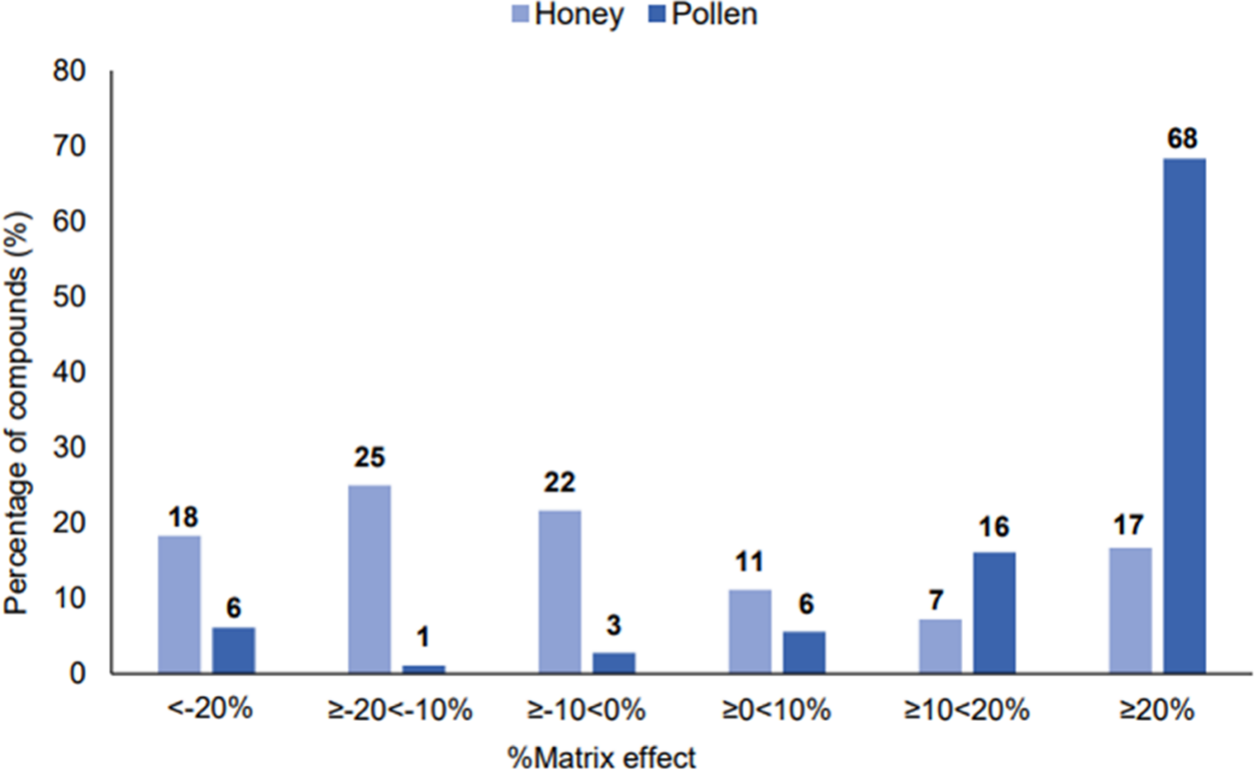
Effect of the matrices, honey and pollen, on the signal response of 180 pesticides analyzed by GC-MS/MS. The data is presented as a percentage of compounds per percentage within each percentage range of matrix effect, categorized into six ranges from < −20 to >20%.

As part of the validation process, the matrix effect was measured to identify and address any bias in the method. The results of this study indicate that the effect of the matrix is more significant in pollen compared to honey. Specifically, 74.4% of pesticides presented a significant matrix effect in pollen, whereas only 35.0% exhibited a significant matrix effect in honey. When comparing the Mass-Spec signal, an increased signal was predominantly observed in pesticides matched with pollen (68.3%) than in those associated with honey (16.7%) (Figure 3). A significant decrease in the Mass-Spec signal was observed in only 6.1% of the analytes in pollen, while in honey, a significant decrease in the signal was observed in 18.3% of the compounds (Table S5).

The mini-Luke method for 5 g of sample was successfully evaluated using the quantification check sample (No. 804-194) positive for boscalid. Using the modified method, sample 804–194 presented a boscalid concentration of 0.015 mg/kg and an *R*^2^ value of 0.991. The percentage of recovery for boscalid was 99.5%. The concentration of the pesticide detected was below the MRL of 0.150 mg/kg set by the EU, and the LOQ for this compound was determined at 0.010 mg/kg.

### Pesticide Detection in Real Samples

The modified and validated NL method was used to determine the levels of pesticides bees were exposed to on the island of Ireland from 2020 to 2021. A representative group of 191 samples, including 92 honey and 99 pollen, was collected by beekeepers as part of the National Apiculture Program and analyzed utilizing this multiresidue analysis for the detection of 346 compounds. Although the method was optimized for 5 g of sample, we were able to accurately analyze samples where the volume ranged from 3.26 to 5 g. Samples smaller than 5 g were utilized when a bigger portion was not available.

Among the tested samples, 22 (11.5%) were identified as positives for containing pesticide residues above the limit of quantification set at the lower calibration level. The concentrations of pesticides ranged from 0.010 mg/kg to 0.158 mg/kg. The most commonly found pesticides in honey and pollen samples were the insecticide-acaricides propargite, coumaphos, and tau-fluvalinate; the fungicide boscalid; and the herbicide MCPA (Table 2). Propargite was the most prevalent residue found in Irish beekeeping resources (pollen and honey) being present in 50.0% of the positive samples either as a single residue or in combination with other analytes. Propargite was found in concentrations ranging from 0.013 to 0.158 mg/kg, with the highest concentration present in pollen. In honey, the highest concentration of this insecticide-acaricide was detected at 0.048 mg/kg.

**Table 2:** Pesticides Detected in Real Honey and Pollen Samples Collected across the Island of Ireland^a^

pesticide	class	no. of positive samples	AVG. concentration (mg/kg)	frequency of detection (%) *N* = 22
2,4-D	herbicide	1	0.080	4.55
azoxystrobin	fungicide	1	0.022	4.55
boscalid	fungicide	2	0.019	9.09
coumaphos	insecticide-acaricide	1	0.022	4.55
cyprodinil	fungicide	1	0.056	4.55
fludioxonil	fungicide	1	0.047	4.55
MCPA	herbicide	3	0.052	13.64
propargite	insecticide-acaricide	7	0.078	31.82
quizalofop	herbicide	1	0.036	4.55
tau-fluvalinate	insecticide-acaricide	2	0.047	9.09
trifluralin	herbicide	1	0.011	4.55
**boscalid**	**fungicide**	**1**	**0.010**	**4.55**
**DDAC**	**fungicide**	**1**	**0.010**	**4.55**
**propargite**	**insecticide-acaricide**	**4**	**0.029**	**18.18**

a The average (AVG) represents the concentration of positive samples per pesticide. The limit of quantification (LOQ) was established at 0.019 mg/kg for the herbicide 2,4-D, while for the remaining compounds, it was set at 0.010 mg/kg. Pesticides found in honey are highlighted in **bold**, while the remaining data corresponds to analytes detected in pollen.

Single residues were found in all six positive honey samples and in 13 positive pollen samples, while multiple residues were found in 3 pollen test portions. The most prevalent combination of pesticides observed was a mixture of insecticide-acaricides. For example, one pollen sample exhibited residues of three insecticide-acaricides (propargite, fluvalinate-tau and coumaphos) at concentrations of 0.118, 0.028, and 0.022 mg/kg, respectively. In a different pollen sample, a combination of propargite at 0.158 mg/kg and fluvalinate-tau at 0.065 mg/kg was detected. Another combination of multiresidues present in pollen included a blend of fungicides (cyprodinil and fludioxonil) at 0.056 and 0.047 mg/kg, respectively.

**Figure 4:**
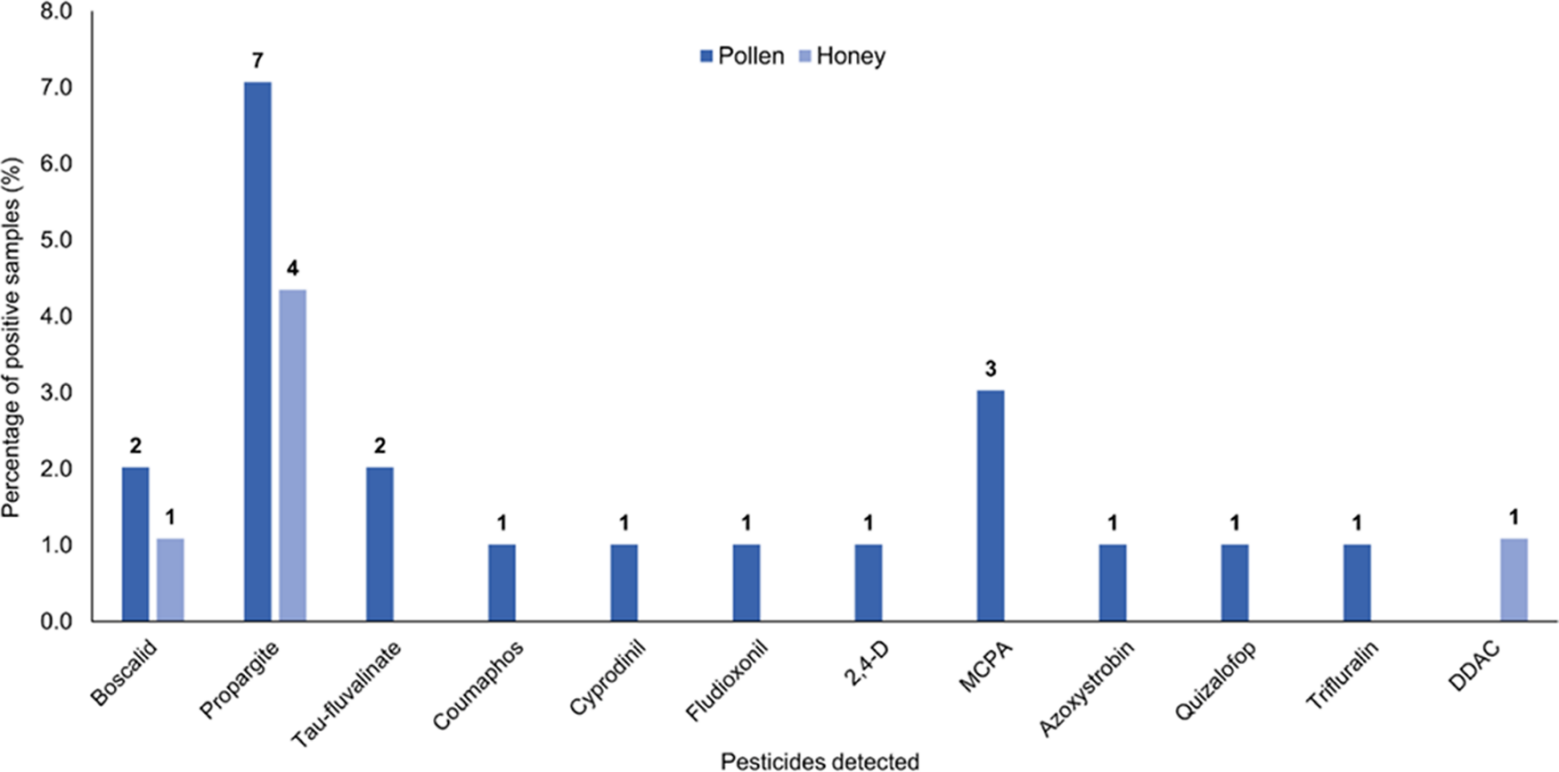
Percentage of positive samples of pollen and honey collected across the island of Ireland.

Positive pollen samples contained a higher number of analytes, with 11 pesticides detected, in comparison to honey samples where only three compounds (propargite, boscalid, and DDAC) were found (Figure 4). None of the residues detected in honey exceeded the maximum residue limits (MRLs) established by Regulation (EU) No 283/2013, which sets the limits at 0.150 mg/kg for boscalid and as 0.050 mg/kg for the remaining analytes in honey and other apiculture products.^11^ These regulations may not apply for residues found in pollen samples.^11,36^

The modified method presented reliable results in terms of precision and accuracy when applied in real pollen and honey samples. Analytes presented in these samples showed good recovery fluctuating from 64.6 to 108.8%. These results highlight the consistent performance of the modified method in obtaining reliable recovery rates for both pollen and honey samples across the two analytical techniques GC-MS/MS and UHPLC-MS.

## Discussion

### Validation of the Extraction Method

The accurate detection of pesticides in honey and pollen is essential for determining the level of pesticides that honey bees and other pollinators are exposed to in the environment and their colonies.^10,27^ Adjusting the NL method enabled us to successfully detect pesticides in over 20 small samples (5 g) each of honey and pollen. The modified NL method showed consistent results in terms of accuracy, precision, and pesticide detection, and aligned with the validation criteria and findings of previous studies.^16,20,29,37^

Previous studies have focused on adapting different extraction methods to improve the efficiency of pesticide detection in complex matrices such as honey.^38^ As an example, in a recent study, the QuEChERS extraction method was optimized for detecting pesticides in honey using GC-MS/MS and LC-MS/MS. In that study, the addition of sorbents during the clean-up step enabled a consistent recovery of over 70% for the majority of the pesticides detected in this matrix.^38^ Other methods have also aimed to enhance precision measured through recovery experiments. For example, Česnik et al. developed multiresidue analytical methods and validated these methods for detecting pesticides in organic and commercial honey samples by GC-MS/MS and LC-MS/MS. Some of these methods involved the use of solvents such as acetone (40 mL), dichloromethane (80 ml), and petroleum ether (80 ml), similar to the NL method. These methods achieved acceptable recovery rates ranging from 70 to 120%, and demonstrated good linearity, with GC achieving a linearity range of 0.960–0.988 and LC ranging from 0.991 to 0.999.^12^ These results align with our study, where over 84% of the compounds extracted from honey samples using the modified NL method and analyzed by GC-MS/MS and UHPLC-MS were successfully validated. Further, our analyses exhibited R^2^ values ranging from 0.956 to 0.999 in GC-MS/MS and from 0.950 to 0.998 in the UHPLC-MS, demonstrating the reliability of the miniaturized method developed here.

We also found that reducing the volume of solvents in the NL method had no adverse effects on the extraction method. Similar findings have been reported recently for the analysis of oranges and lettuce using GC-MS/MS and liquid chromatography–mass spectrometry (LC-MS/MS), where reducing the volume of solvents to 10 mL resulted in good recovery for the majority of the pesticides (approx. 170 out of 175 analytes).^16^ In our study, we reduced the volume of solvents from 30 to 10 mL and the sample weight from 15 to 5 g, achieving good recovery. Here, approximately 80% of the pesticides analyzed by GC-MS/MS and UHPLC-MS at 10 and 100 μg/kg achieved recovery above 80% (Figure 2, Table S5).

The matrix effect in a multiresidue analysis can impact trueness and lead to poor quantification of specific compounds. An accurate analysis of this parameter allows for mitigation of its effects and more accurate quantification, reducing misinterpretation of positive results. Our study revealed that the matrix effect is more significant in pollen than in honey, with over 60% of compounds showing a significant matrix effect in the pollen matrix. Interestingly, some compounds were found to have opposite signal responses in honey versus pollen. We found that propargite and coumaphos showed decreased signals in the honey matrix, but increased signals in pollen. Conversely, bitertanol-II and spirodiclofen displayed decreased signals in pollen but increased in honey. Interestingly, the signals of cyhalothrin-lambda and fludioxonil, and other 80 compounds were unaffected in honey but showed a significant increase in the pollen matrix. On the other hand, 18 compounds including boscalid, tau-fluvalinate, and prochloraz had no significant matrix effect in pollen but showed decreased response in honey. A few compounds, such as dimethoate and methamidophos, had similar responses in both matrices with increased signal response in honey and pollen. These findings highlight the need for using matrix-matched standards to compensate for the matrix effect in quantification.

Previous studies have demonstrated that the signals of certain compounds such as cyhalothrin-lambda, fludioxonil, propargite, and coumaphos are unaffected by honey when using the QuEChERS extraction method.^38^ However, our study reveals a different outcome: we show that the signal response of compounds like propargite and coumaphos in honey decreased when using the NL method for extraction. This suggests that the signal response is influenced by the extraction method as well as the matrix. In addition, it has been suggested that matrix effects are specific to the pesticide–matrix combination, and variations in response can be attributed to the matrix composition, which can be further influenced by sample composition and geographical distribution.^39^ Additional research in this field will contribute to a better understanding of these interactions as well as to the pesticide–matrix relationship.

The modified NL method demonstrated good performance in the analysis of a honey sample with known residue concentration. In the case of honey sample 804–194, the standard extraction method resulted in a boscalid concentration of 0.018 mg/kg. However, when the modified method was utilized, the detected boscalid concentration was 16.7% lower (0.015 mg/kg). This difference is not considered to be significant.^29^

Our findings demonstrate that the modified NL method has been validated as it meets the criteria defined in SANTE 11312/202. It means this method is suitable for the analysis of a wide range of pesticides in honey and pollen and has been shown to be robust when used to analyze pollen and honey by GC-MS/MS and UHPLC-MS. We also demonstrate that the NL method is a versatile extraction method that can be adapted to be used for the analysis of complex matrices such as honey and pollen. Similarly, Gaweł et al.^38^ suggested a miniaturization of the mini-Luke method for the analysis of organic compounds such as Tymol and single residues such as amitraz for the detection of pesticides in honey. Lozano et al.^16^ recommended a miniaturization of this method, with a more environmentally friendly and cost-effective approach, for routine analysis of fruits and vegetables.

We suggest that the miniaturized method validated here can be considered as a useful monitoring tool for determining the presence of pesticides in honey bee colony resources particularly, honey and pollen, and for evaluating the pesticide levels that managed bees and wild pollinators are exposed to.

### Application of the Method to Real Samples

The samples collected and analyzed as part of this study have been used to evaluate the exposure of honey bees and other pollinators to pesticides across the island of Ireland. The extraction method validated here was used for the analysis of honey bee colony resources collected in Irish apiaries. The analysis of a representative group of 191 samples of honey and pollen revealed that 88.5% of the assessed apiaries were free of pesticides, while 11.5% showed the presence of pesticides. Among the residues detected in honey and pollen, 41.7% were fungicides, followed by herbicides (33.3%) and insecticide-acaricides (25.0%), distributed across the entire island. In addition, 86.4% of the positive samples exhibited single residues, while the remaining 13.6% contained multiple residues, either in the form of insecticide-acaricide blends (i.e., propargite, tau-fluvalinate, and coumaphos; and propargite with tau-fluvalinate) or fungicide combinations (fludioxonil and cyprodinil). Multiple residues were predominantly found in pollen rather than in honey.

Propargite residues were the most prevalent in honey and pollen samples, either as a single residue or in combinations with other insecticide-acaricides. It was present in 50.0% of the positive samples, detected in 11 samples (7 pollen and 4 honey). The presence of propargite inside honey bee colonies may be attributed to foraging worker bees bringing contaminated resources into the colony.^7^ Previous studies have indicated that propargite has minimal to no effect on untargeted insects when applied within regulatory guidelines.^40^ However, it has been reported that in^45^ association with other compounds, propargite can have a higher toxicity and lethal consequences for bees, especially the alfalfa leaf-cutting bee (*Megachile rotundata*), which is thought to be more susceptible to insecticides than honey bees.^41^ Due to the fact that propargite is a lipophilic compound, it can also be transferred into the colony resources from contaminated wax, as has been demonstrated with other pesticides such as coumaphos.^42^ To our knowledge, this is the first time the insecticide-acaricide propargite has been detected in these matrices in Ireland. Propargite was previously reported in wax foundation in the US in 2009 and 2011,^43^ and in beeswax in Belgium in 2015.^7^

The insecticide-acaricide tau-fluvalinate was present in two pollen samples at concentrations of 0.028 mg/kg and 0.065 mg/kg, while in the same category, coumaphos was found in one sample at 0.022 mg/kg. The presence of veterinary products such as coumaphos and tau-fluvalinate is primarily attributed to in-hive treatments, but indirect contamination through previously contaminated recycled wax can also be a contributing factor.^44^ Coumaphos and tau-fluvalinate are pesticides commonly used to control *Varroa* mites, and residues of these products are frequently found in beekeeping-related matrices.^45,46^ For instance, Mullin et al.,^51^ reported that 47% of tested samples (beeswax and pollen) in North American apiaries contained both acaricides. Coumaphos and tau-fluvalinate are pesticides with low toxicity for bees.^45^ However, it appears that the presence of both compounds in the hive at sublethal levels creates an interaction between compounds (synergism) that can modify the detoxification metabolism in honey bees.^45^ The synergistic interaction instigates a competition between active ingredients for accessing the enzyme P450 involved in detoxication.^45^ It has been reported that this synergistic interaction can occur in managed bees when coumaphos and tau-fluvalinate are applied at the manufacturer’s recommended dosage, and its impact depends on the caste and age of bees.^45^ For example, larvae are likely more susceptible to the synergistic interaction of insecticides because they are continuously in contact with beeswax.^45^ Importantly, it has been reported that the presence of both compounds in beeswax does not affect the emergence time of queens, but can compromise the queen’s fertility by reducing viability and sperm count in the queen’s spermatheca.^47^ In addition, it has been estimated that both acaricides, tau-fluvalinate and coumaphos, can persist for 5 years in beeswax,^48^ which suggests a continuous and persistent risk for bees. Tau-fluvalinate and coumaphos are authorized by the EU and both are considered bee-safe when they are applied under good agricultural practice.^49,50^

The fungicides cyprodinil and fludioxonil were found in one pollen sample at 0.056 and 0.047 mg/kg, respectively. Interestingly, cyprodinil has been previously reported in other studies within this matrix at different concentration levels ranging from 5.3 to 344 ppb, and it has been described as potentially synergistic.^51^ In addition, the fungicide boscalid was found in 3 samples (2 pollen and 1 honey) at concentration levels ranging from 0.010 to 0.019 mg/kg. Studies have indicated that chlorothalonil and boscalid are the most common fungicides found in pollen.^51^ Although boscalid was previously considered safe for adult honey bees,^52^ it has been detected in incidents resulting in significant honey bee mortality.^53,54^ Further research has indicated that boscalid can negatively affect the development of honey bees and their brood leading to high mortality rates when applied in larger doses in the field.^55,56^ In general, the presence of fungicides in pollen is attributed to worker bees collecting pollen from flowers that have been directly sprayed with fungicides in farmlands, home gardens, or roadside meadows.^51,57^ Fungicides can also indirectly enter pollen through spray-generated dust that is transported by wind gusts, contaminating untreated flowers, soil, and water sources.^57^

The herbicide MCPA was detected in three pollen samples ranging from 0.027 to 0.043 mg/kg. In contrast, the herbicide 2,4-D was found in one pollen sample at a concentration of 0.080 mg/kg. Low concentrations of other herbicides such as quizalofop and trifluralin were also found in pollen. MCPA has been previously detected in honey and honey bees in areas where hive poisoning incidents have been reported.^54^ MCPA and 2,4-D are selective herbicides used to control annual and perennial weeds, such as charlock, wild radish, and dandelion. As systemic herbicides, they can translocate through the plant contaminating pollen and nectar, and can persist in soil for 1–3 months.^58^ The EU has determined that traces of MCPA have low toxicity to honey bees.^59^

A small amount (0.0102 mg/kg) of the biocide active ingredient DDAC (didecyldimethylammonium) was detected in honey, which can be attributed to the use of DDAC-based disinfectants during honey harvesting, and in the food production chain.^60^ Further, according to the literature, residues of this compound can contaminate honey bee colonies when the wood used to construct the beehives has been treated with products containing DDAC.^61^

The detection of pesticides in honey and pollen, which are the main dietary resources for bees, raises concerns about the continuous oral exposure of honey bees to pesticides throughout the island of Ireland. Our findings suggest that 21 apiaries across the island were contaminated with low levels of pesticides. However, the presence of banned analytes such as propargite and other residues exceeding the maximum residue limits (MRLs), where applicable, suggests the occurrence of different pesticide applications in Ireland that can have a negative impact on pollinators. These practices may include incorrect application of agrochemicals, unintentional drift of substances beyond the intended application fields, pesticide applications during blooming seasons when bees are actively foraging, accumulation of pesticide residues in beekeeping materials, or even deliberate application of pesticides to harm honey bee colonies.^62^

In conclusion, we suggest that the modified NL method presented in our study, adapted for small-size samples of honey and pollen, can serve as a valuable tool for identifying specific stress factors contributing to honey bee colony losses and the decline of wild pollinator populations, particularly the presence of pesticides in key beekeeping areas. By detecting the presence of pesticide stressors, we can contribute to protect the health and well-being of honey bees and other pollinators by developing mitigation and conservation strategies, as well as ensuring the availability of safe food supplies. These findings emphasize the importance of closely monitoring areas of concern to protect honey bees and preserve the pollinator populations across Ireland and beyond.

## Supporting Information Available

GC-MS/MS and UHPLC instrumental setting parameters (Tables S1–S3), additional experimental data (Tables S4 S5), and screenshots of calibration curves and chromatograms from positive samples (Figures S1–S5) (PDF)

## References

[ref1] Steinhauer N.; Kulhanek K.; Antúnez K.; Human H.; Chantawannakul P.; Chauzat M.-P.; VanEngelsdorp D. Drivers of Colony Losses. Curr. Opin. Insect. Sci. 2018, 26, 142–148. 10.1016/j.cois.2018.02.004. 29764654

[ref2] vanEngelsdorp D.; Evans J. D.; Saegerman C.; Mullin C.; Haubruge E.; Nguyen B. K.; Frazier M.; Frazier J.; Cox-Foster D.; Chen Y.; Underwood R.; Tarpy D. R.; Pettis J. S. Colony Collapse Disorder: A Descriptive Study. PLoS One 2009, 4, e6481 10.1371/journal.pone.0006481. 19649264 PMC2715894

[ref3] Portus R. An Ecological Whodunit: The Story of Colony Collapse Disorder. Soc. Anim. 2020, 31, 242–260. 10.1163/15685306-BJA10026.

[ref4] Botías C.; David A.; Horwood J.; Abdul-Sada A.; Nicholls E.; Hill E.; Goulson D. Neonicotinoid Residues in Wildflowers, a Potential Route of Chronic Exposure for Bees. Environ. Sci. Technol. 2015, 49, 12731–12740. 10.1021/acs.est.5b03459. 26439915

[ref5] Zioga E.; Kelly R.; White B.; Stout J. C. Plant Protection Product Residues in Plant Pollen and Nectar: A Review of Current Knowledge. Environ. Res. 2020, 189, 109873 10.1016/j.envres.2020.109873. 32795671

[ref6] Tanner G.; Czerwenka C. LC-MS/MS Analysis of Neonicotinoid Insecticides in Honey: Methodology and Residue Findings in Austrian Honeys. J. Agric. Food Chem. 2011, 59, 12271–12277. 10.1021/jf202775m. 22026460

[ref7] Ravoet J.; Reybroeck W.; de Graaf D. C. Pesticides for Apicultural and/or Agricultural Application Found in Belgian Honey Bee Wax Combs. Bull. Environ. Contam. Toxicol. 2015, 94, 543–548. 10.1007/s00128-015-1511-y. 25749505 PMC4391733

[ref8] Mommaerts V.; Reynders S.; Boulet J.; Besard L.; Sterk G.; Smagghe G. Risk Assessment for Side-Effects of Neonicotinoids against Bumblebees with and without Impairing Foraging Behavior. Ecotoxicology 2010, 19, 207–215. 10.1007/s10646-009-0406-2. 19757031

[ref9] van der Sluijs J. P.; Simon-Delso N.; Goulson D.; Maxim L.; Bonmatin J.-M.; Belzunces L. P. Neonicotinoids, Bee Disorders and the Sustainability of Pollinator Services. Curr. Opin. Environ. Sustainability 2013, 5, 293–305. 10.1016/j.cosust.2013.05.007.

[ref10] Niell S.; Jesús F.; Pérez C.; Mendoza Y.; Díaz R.; Franco J.; Cesio V.; Heinzen H. QuEChERS Adaptability for the Analysis of Pesticide Residues in Beehive Products Seeking the Development of an Agroecosystem Sustainability Monitor. J. Agric. Food Chem. 2015, 63, 4484–4492. 10.1021/acs.jafc.5b00795. 25880394

[ref11] EURL (EU Reference Laboratories). Technical Guidelines for Determining the Magnitude of Pesticide Residues in Honey and Setting Maximum Residue Levels in Honey SANTE/11956/2016, 2018.

[ref12] Baša Česnik H.; Kmecl V.; Velikonja Bolta Š. Pesticide and Veterinary Drug Residues in Honey - Validation of Methods and a Survey of Organic and Conventional Honeys from Slovenia. Food Addit. Contam.: Part A 2019, 36, 1358–1375. 10.1080/19440049.2019.1631492. 31287377

[ref13] Narenderan S. T.; Meyyanathan S. N.; Babu B. Review of Pesticide Residue Analysis in Fruits and Vegetables. Pre-Treatment, Extraction and Detection Techniques. Food Res. Int. 2020, 133, 109141 10.1016/j.foodres.2020.109141. 32466907

[ref14] Sulaiman N. S.; Rovina K.; Joseph V. M. Classification, Extraction and Current Analytical Approaches for Detection of Pesticides in Various Food Products. J. Consum. Prot. Food Saf. 2019, 14, 209–221. 10.1007/s00003-019-01242-4.

[ref15] Pihlström T.; Blomkvist G.; Friman P.; Pagard U.; Österdahl B.-G. Analysis of Pesticide Residues in Fruit and Vegetables with Ethyl Acetate Extraction Using Gas and Liquid Chromatography with Tandem Mass Spectrometric Detection. Anal. Bioanal. Chem. 2007, 389, 1773–1789. 10.1007/s00216-007-1425-6. 17609934

[ref16] Lozano A.; Kiedrowska B.; Scholten J.; de Kroon M.; de Kok A.; Fernández-Alba A. R. Miniaturisation and Optimisation of the Dutch Mini-Luke Extraction Method for Implementation in the Routine Multi-Residue Analysis of Pesticides in Fruits and Vegetables. Food Chem. 2016, 192, 668–681. 10.1016/j.foodchem.2015.07.065. 26304397

[ref17] Rejczak T.; Tuzimski T. A Review of Recent Developments and Trends in the QuEChERS Sample Preparation Approach. Open Chem 2015, 13, 980–1010. 10.1515/chem-2015-0109.

[ref18] European Union Reference Laboratory for Pesticide Residues - Fruits and Vegetables (EURL-FV). Dutch Mini-Luke (“NL-”) Extraction Method Followed by LC and GC-MS/MS for Multiresidue Analysis of Pesticides in Fruits and Vegetables, 2014.

[ref19] Guan H.; Brewer W. E.; Garris S. T.; Craft C.; Morgan S. L. Multiresidue Analysis of Pesticides in Fruits and Vegetables Using Disposable Pipette Extraction (DPX) and Micro-Luke Method †. J. Agric. Food Chem. 2010, 58, 5973–5981. 10.1021/jf903448w. 20218611

[ref20] Garvey J.; Walsh T.; Devaney E.; King T.; Kilduff R. Multi-Residue Analysis of Pesticide Residues and Polychlorinated Biphenyls in Fruit and Vegetables Using Orbital Ion Trap High-Resolution Accurate Mass Spectrometry. Anal. Bioanal. Chem. 2020, 412, 7113–7121. 10.1007/s00216-020-02844-w. 32749509

[ref21] Elshabrawy M. S.; Khorshid M. A.; Hamdy Abdelwahed M.; Abo-Aly M. M. Optimization and Evaluation of Four Multi-Residue Methods for the Determination of Pesticide Residues in Orange Oil Using LC-MS/MS and GC-MS/MS: A Comparative Study. Int. J. Environ. Anal. Chem. 2021, 1–18. 10.1080/03067319.2021.1921761.

[ref22] Meghesan-Breja A.; Cimpoiu C.; Hosu A. Identification and Quantification of Some Pesticide Metabolites from Vegetables by GC-TOF-MS and LC-MS-QQQ. Stud. Univ. Babeş-Bolyai Chem. 2017, 62, 19–34. 10.24193/subbchem.2017.3.02.

[ref23] Sawyer L. D. The Luke et al. Method for Determining Multipesticide Residues in Fruits and Vegetables: Collaborative Study. J. - Assoc. Off. Anal. Chem. 1985, 68, 64–71. 10.1093/jaoac/68.1.64. 3980415

[ref24] Vickneswaran M.; Carolan J. C.; White B. Simultaneous Determination of Pesticides from Soils: A Comparison between QuEChERS Extraction and Dutch Mini-Luke Extraction Methods. Anal. Methods 2021, 13, 5638–5650. 10.1039/D1AY01248G. 34787125

[ref25] Lozano A.; Hernando M. D.; Uclés S.; Hakme E.; Fernández-Alba A. R. Identification and Measurement of Veterinary Drug Residues in Beehive Products. Food Chem. 2019, 274, 61–70. 10.1016/j.foodchem.2018.08.055. 30372985

[ref26] Beyer M.; Lenouvel A.; Guignard C.; Eickermann M.; Clermont A.; Kraus F.; Hoffmann L. Pesticide Residue Profiles in Bee Bread and Pollen Samples and the Survival of Honeybee Colonies—a Case Study from Luxembourg. Environ. Sci. Pollut. Res. 2018, 25, 32163–32177. 10.1007/s11356-018-3187-4. 30220063

[ref27] Kiljanek T.; Niewiadowska A.; Małysiak M.; Posyniak A. Miniaturized Multiresidue Method for Determination of 267 Pesticides, Their Metabolites and Polychlorinated Biphenyls in Low Mass Beebread Samples by Liquid and Gas Chromatography Coupled with Tandem Mass Spectrometry. Talanta 2021, 235, 122721 10.1016/j.talanta.2021.122721. 34517589

[ref28] Guide to Achieving Reliable Quantitative LC-MS Measurements, RSC Analytical Methods Committee, 1st ed.; Sargent M., Ed., 2013.

[ref29] EURL (EU Reference Laboratories). Analytical Quality Control and Method Validation Procedures for Pesticide Residues Analysis in Food and Feed SANTE/11312/2021, European Commision - EURL website, 2021. https://www.eurl-pesticides.eu/docs/public/tmplt_article.asp?CntID=727&LabID=100&Lang=EN.

[ref30] Lister A. S. Validation of HPLC Methods in Pharmaceutical Analysis. In Separation Science and Technology; Ahuja S.; Dong M. W., Eds.; Academic Press, 2005; pp 191–217 10.1016/S0149-6395(05)80051-0.

[ref31] Rahman Md. M.; Abd El-Aty A. M.; Shim J.-H. Matrix Enhancement Effect: A Blessing or a Curse for Gas Chromatography?—A Review. Anal. Chim. Acta 2013, 801, 14–21. 10.1016/j.aca.2013.09.005. 24139570

[ref32] Steiner D.; Krska R.; Malachová A.; Taschl I.; Sulyok M. Evaluation of Matrix Effects and Extraction Efficiencies of LC–MS/MS Methods as the Essential Part for Proper Validation of Multiclass Contaminants in Complex Feed. J. Agric. Food Chem. 2020, 68, 3868–3880. 10.1021/acs.jafc.9b07706. 32125845 PMC7205385

[ref33] Lehotay S. J.; Son K. A.; Kwon H.; Koesukwiwat U.; Fu W.; Mastovska K.; Hoh E.; Leepipatpiboon N. Comparison of QuEChERS Sample Preparation Methods for the Analysis of Pesticide Residues in Fruits and Vegetables. J. Chromatogr. A 2010, 1217, 2548–2560. 10.1016/j.chroma.2010.01.044. 20144460

[ref34] Kieliszek M.; Piwowarek K.; Kot A. M.; Błażejak S.; Chlebowska-Śmigiel A.; Wolska I. Pollen and Bee Bread as New Health-Oriented Products: A Review. Trends Food Sci. Technol. 2018, 71, 170–180. 10.1016/j.tifs.2017.10.021.

[ref35] Anderson K. E.; Carroll M. J.; Sheehan T.; Mott B. M.; Maes P.; Corby-Harris V. Hive-stored Pollen of Honey Bees: Many Lines of Evidence Are Consistent with Pollen Preservation, Not Nutrient Conversion. Mol. Ecol. 2014, 23, 5904–5917. 10.1111/mec.12966. 25319366 PMC4285803

[ref36] European Council Directive. Regulation (EC) No 1107/2009 of the European Parliament and of the Council. https://www.legislation.gov.uk/eur/2009/1107/data.xht?view=snippet&wrap=true, 2009.

[ref37] Mol H. G. J.; Plaza-Bolaños P.; Zomer P.; de Rijk T. C.; Stolker A. A. M.; Mulder P. P. J. Toward a Generic Extraction Method for Simultaneous Determination of Pesticides, Mycotoxins, Plant Toxins, and Veterinary Drugs in Feed and Food Matrixes. Anal. Chem. 2008, 80, 9450–9459. 10.1021/ac801557f. 19072261

[ref38] Gaweł M.; Kiljanek T.; Niewiadowska A.; Semeniuk S.; Goliszek M.; Burek O.; Posyniak A. Determination of Neonicotinoids and 199 Other Pesticide Residues in Honey by Liquid and Gas Chromatography Coupled with Tandem Mass Spectrometry. Food Chem. 2019, 282, 36–47. 10.1016/j.foodchem.2019.01.003. 30711104

[ref39] Vázquez P. P.; Lozano A.; Uclés S.; Ramos M. M. G.; Fernández-Alba A. R. A Sensitive and Efficient Method for Routine Pesticide Multiresidue Analysis in Bee Pollen Samples Using Gas and Liquid Chromatography Coupled to Tandem Mass Spectrometry. J. Chromatogr. A 2015, 1426, 161–173. 10.1016/j.chroma.2015.11.081. 26654830

[ref40] Kumar V.; Sood C.; Jaggi S.; Ravindranath S. D.; Bhardwaj S. P.; Shanker A. Dissipation Behavior of Propargite––an Acaricide Residues in Soil, Apple (Malus Pumila) and Tea (Camellia Sinensis). Chemosphere 2005, 58, 837–843. 10.1016/j.chemosphere.2004.06.032. 15621197

[ref41] Johansen C. A.; Mayer D. F.; Eves J. D.; Kious C. W. Pesticides and Bees 1. Environ. Entomol. 1983, 12, 1513–1518. 10.1093/ee/12.5.1513.

[ref42] Tremolada P.; Bernardinelli I.; Colombo M.; Spreafico M.; Vighi M. Coumaphos Distribution in the Hive Ecosystem: Case Study for Modeling Applications. Ecotoxicology 2004, 13, 589–601. 10.1023/B:ECTX.0000037193.28684.05. 15526863

[ref43] Ostiguy N.; Drummond F. A.; Aronstein K.; Eitzer B.; Ellis J. D.; Spivak M.; Sheppard W. S. Honey Bee Exposure to Pesticides: A Four-Year Nationwide Study. Insects 2019, 10, 13 10.3390/insects10010013. 30626027 PMC6359572

[ref44] Department for Environment Food and Rural Affairs, D. The Expert Committee on Pesticide Residues in Food (PRiF). Anual Report, 2019, 2019.

[ref45] Johnson R. M.; Pollock H. S.; Berenbaum M. R. Synergistic Interactions Between In-Hive Miticides in Apis Mellifera. J. Econ. Entomol. 2009, 102, 474–479. 10.1603/029.102.0202. 19449624

[ref46] Wilmart O.; Legrève A.; Scippo M.-L.; Reybroeck W.; Urbain B.; de Graaf D. C.; Steurbaut W.; Delahaut P.; Gustin P.; Nguyen B. K.; Saegerman C. Residues in Beeswax: A Health Risk for the Consumer of Honey and Beeswax?. J. Agric. Food Chem. 2016, 64, 8425–8434. 10.1021/acs.jafc.6b02813. 27741395

[ref47] Rangel J.; Tarpy D. R. The Combined Effects of Miticides on the Mating Health of Honey Bee (Apis Mellifera L.) Queens. J. Apic. Res. 2015, 54, 275–283. 10.1080/00218839.2016.1147218.

[ref48] Bogdanov S. Beeswax: Quality Issues Today. Bee World 2004, 85, 46–50. 10.1080/0005772X.2004.11099623.

[ref49] Brancato A.; Brocca D.; Carrasco Cabrera L.; De Lentdecker C.; Erdos Z.; Ferreira L.; Greco L.; Jarrah S.; Kardassi D.; Leuschner R.; Lostia A.; Lythgo C.; Medina P.; Miron I.; Molnar T.; Pedersen R.; Reich H.; Sacchi A.; Santos M.; Stanek A.; Sturma J.; Tarazona J.; Theobald A.; Vagenende B.; Villamar-Bouza L. Review of the Existing Maximum Residue Levels for Tau-fluvalinate According to Article 12 of Regulation (EC) No 396/2005. EFSA J. 2018, 16, e05475 10.2903/j.efsa.2018.5475. 32625757 PMC7009756

[ref50] Setting of Maximum Residue Levels for Amitraz, Coumaphos, Flumequine, Oxytetracycline, Permethrin and Streptomycin in Certain Products of Animal Origin. EFSA J. 2016, 14, e04570 10.2903/j.efsa.2016.4570.

[ref51] Mullin C. A.; Frazier M.; Frazier J. L.; Ashcraft S.; Simonds R.; VanEngelsdorp D.; Pettis J. S. High Levels of Miticides and Agrochemicals in North American Apiaries: Implications for Honey Bee Health. PLoS One 2010, 5, e9754 10.1371/journal.pone.0009754. 20333298 PMC2841636

[ref52] Institute, E. P. A. Environmental Fate and Ecological Risk Assessment for Boscalid New Use on Rapeseed, Including Canola (Seed Treatment), 2010.

[ref53] Kasiotis K. M.; Anagnostopoulos C.; Anastasiadou P.; Machera K. Pesticide Residues in Honeybees, Honey and Bee Pollen by LC–MS/MS Screening: Reported Death Incidents in Honeybees. Sci. Total Environ. 2014, 485–486, 633–642. 10.1016/j.scitotenv.2014.03.042. 24747255

[ref54] Kiljanek T.; Niewiadowska A.; Semeniuk S.; Gaweł M.; Borzęcka M.; Posyniak A. Multi-Residue Method for the Determination of Pesticides and Pesticide Metabolites in Honeybees by Liquid and Gas Chromatography Coupled with Tandem Mass Spectrometry—Honeybee Poisoning Incidents. J. Chromatogr. A 2016, 1435, 100–114. 10.1016/j.chroma.2016.01.045. 26830634

[ref55] Fisher A.; DeGrandi-Hoffman G.; Smith B. H.; Johnson M.; Kaftanoglu O.; Cogley T.; Fewell J. H.; Harrison J. F. Colony Field Test Reveals Dramatically Higher Toxicity of a Widely-Used Mito-Toxic Fungicide on Honey Bees (Apis Mellifera). Environ. Pollut. 2021, 269, 115964 10.1016/j.envpol.2020.115964. 33261965

[ref56] Simon-Delso N.; San Martin G.; Bruneau E.; Hautier L.; Medrzycki P. Toxicity Assessment on Honey Bee Larvae of a Repeated Exposition of a Systemic Fungicide, Boscalid. Bulletin of Toxicology 2017, 70, 83–89.

[ref57] Friedle C.; Wallner K.; Rosenkranz P.; Martens D.; Vetter W. Pesticide Residues in Daily Bee Pollen Samples (April–July) from an Intensive Agricultural Region in Southern Germany. Environ. Sci. Pollut. Res. 2021, 28, 22789–22803. 10.1007/s11356-020-12318-2. PMC8113304 33432407

[ref58] Zimdahl R. L. 2,4-D: An Herbicide. In Six Chemicals That Changed Agriculture; Elsevier, 2015; pp 89–113 10.1016/B978-0-12-800561-3.00006-7.

[ref59] Lewis K. A.; Tzilivakis J.; Warner D. J.; Green A. An International Database for Pesticide Risk Assessments and Management. Hum. Ecol. Risk Assess. 2016, 22, 1050–1064. 10.1080/10807039.2015.1133242.

[ref60] Jäger J. E. Chemical Hazards in Foods of Animal Origin. In ECVPH Food safety assurance; Smulders F. J. M.; Rietjens I. M. C. M.; Rose M., Eds.; Wageningen Academic Publishers: The Netherlands, 2019; Vol. 7 10.3920/978-90-8686-877-3.

[ref61] Knizner S. Didecyl Dimethyl Ammonium Chloride (DDAC) Final Work Plan Registration Review: Initial Docket Case Number 3003; United States Environmental Protection Agency: Washington DC, 2017. p 126. https://www3.epa.gov/pesticides/chem_search/reg_actions/reregistration/red_G-6_3-Aug-06.pdf.

[ref62] Kasiotis K. M.; Zafeiraki E.; Manea-Karga E.; Anastasiadou P.; Machera K. Pesticide Residues and Metabolites in Greek Honey and Pollen: Bees and Human Health Risk Assessment. Foods 2023, 12, 706 10.3390/foods12040706. 36832781 PMC9955768

