# Supplementary material for: Analysis of Pesticide Levels in Honey and Pollen from Irish Honey Bee Colonies Using a Modified Dutch Mini-Luke Method with Gas and Liquid Chromatography–Tandem Mass Spectrometry Detection
Source: J Agric Food Chem. 2023 Aug 16;71(34):12657–67. doi: 10.1021/acs.jafc.3c02250 (PMC10472503; doi:10.1021/acs.jafc.3c02250)
Supplement: Supplementary file 1 — jf3c02250_si_001.pdf [file jf3c02250_si_001.pdf]

**Supporting Information for:**

# Analysis of pesticide levels in honey and pollen from Irish honey bee colonies using a modified Dutch mini-Luke method with gas and liquid chromatography-tandem mass spectrometry detection

Marcela A. Díaz*[1, i], Darren P. O'Connell[1, ii], Seana Jordan[2], Catriona O'Connor[2], Paul Martin[2], Julia C. Jones[1, iii], Jim Garvey*[2, iv]

[1] School of Biology and Environmental Science, University College Dublin, Belfield, Dublin, D04 N2E5, Ireland

[2] Food Chemistry Division, Department of Agriculture, Food and The Marine, Celbridge, W23 X3PH, Ireland

ORCID ID: [i]0000-0002-0674-6386, [ii]0000-0001-9331-8189, [iii] 0000-0002-3557-1941, [iv]0000-0002-9267-5149

*Corresponding authors:
Marcela A. Díaz, marcela.diazrivadeneira@ucdconnect.ie
Jim Garvey, Jim.Garvey@agriculture.gov.ie

**Supporting Information**

**Table – S1.** GC-MS/MS Temperature program

| Program | Rate °C/min | Temperature (°C) | Hold time (min) | Run time (min) |
|---|---|---|---|---|
| Initial | - | 80 | 2 | 2 |
| Ramp 1 | 25 | 150 | 0 | 4.8 |
| Ramp 2 | 3 | 200 | 0 | 21.5 |
| Ramp 3 | 8 | 280 | 10 | 41.5 |

**Table – S2.** UHPLC-MS Gradient

| Time (min) | % A | % B |
|---|---|---|
| 0.0 | 95.0 | 5.0 |
| 0.8 | 60.0 | 40.0 |
| 3.5 | 60.0 | 40.0 |
| 5.0 | 40.0 | 60.0 |
| 6.0 | 40.0 | 60.0 |
| 12.0 | 35.0 | 65.0 |
| 17.0 | 0.0 | 100.0 |
| 20.0 | 0.0 | 100.0 |

**Table – S3.** UHPLC-MS Parameters

| Parameter | Value |
|---|---|
| Gas temperature | 120 $^{o}$C |
| Gas flow | 20 l/min |
| Nebuliser pressure | 35 psi |
| Sheath gas temperature | 350 $^{o}$C |
| Sheath gas flow | 12 l/min |
| Nozzle voltage | 300 V |
| Chamber current | 0.2 mA |

**Table – S4.** Transitions (T) or ion pairs detected by GC-MS/MS and UHPLC-MS

| | | T1 | | T2 | |
|---|---|---|---|---|---|
| | | Precursor ion (*m/z*) | Product ion (*m/z*) | Precursor ion (*m/z*) | Product ion (*m/z*) |
| | | **GC Compounds** | | | |
| 1 | 1,4-Dimethylnapthalene | 156 | 141 | 156 | 128 |
| 2 | 4,4-Dichlorobenzophenone | 139 | 111 | 139 | 75.1 |
| 3 | Acephate | 136 | 42 | 136 | 94 |
| 4 | Acrinathrin | 289 | 93 | 181 | 152 |
| 5 | Aldrin | 263 | 193 | 263 | 191 |
| 6 | Anthraquinone | 208.1 | 180.1 | 208.1 | 152.1 |
| 7 | Azinphos-methyl | 160.1 | 132.1 | 160.1 | 77.1 |
| 8 | Azoxystrobin | 344 | 329 | 387.8 | 345.2 |
| 9 | Bifenthrin | 181 | 165 | 181 | 166 |
| 10 | Binapacryl | 82.9 | 55.1 | 82.9 | 39 |
| 11 | Biphenyl | 153.9 | 153.2 | 153.9 | 152.2 |
| 12 | Bitertanol-I | 170 | 115 | 170 | 141 |
| 13 | Bitertanol-II | 170 | 115 | 170 | 141 |
| 14 | Boscalid | 343 | 140 | 343 | 112 |
| 15 | Bromopropylate | 341 | 183 | 341 | 185 |
| 16 | Captafol | 150.9 | 78.9 | 79 | 51 |
| 17 | Captan | 79 | 77 | 79 | 51 |
| 18 | Chlordane-cis | 372.7 | 266.1 | 372.7 | 264.1 |
| 19 | Chlordane-trans | 372.7 | 266.1 | 372.7 | 264.1 |
| 20 | Chlorfenapyr | 247 | 227 | 247 | 197 |
| 21 | Chlorothalonil | 265.9 | 231 | 265.9 | 168 |
| 22 | Chlorpropham | 213 | 171 | 213 | 127 |
| 23 | Chlorpyrifos methyl | 286 | 93 | 286 | 270.9 |
| 24 | Chlorthal-dimethyl | 299 | 221 | 301 | 223 |
| 25 | Cyfluthrin | 163 | 127 | 163 | 91 |
| 26 | Cyhalothrin-lambda | 181.1 | 152.1 | 197 | 141 |
| 27 | Cypermethrin | 181.1 | 152.1 | 181.1 | 127.1 |
| 28 | Cyproconazole | 222 | 125 | 222 | 82 |
| 29 | Deltamethrin | 181 | 152 | 253 | 172 |
| 30 | Diazinon | 304 | 179.1 | 179 | 137.2 |
| 31 | Dicofol | 139 | 111 | 139 | 75 |
| 32 | Dieldrin | 263 | 193 | 263 | 191 |
| 33 | Dimethoate | 142.9 | 111.1 | 142.9 | 125 |
| 34 | Diphenylamine | 169 | 168 | 169 | 167 |
| 35 | Endosulfan-alpha | 240.8 | 206 | 195 | 159 |
| 36 | Endosulfan-beta | 241 | 206 | 195 | 159 |
| 37 | Endosulfan-ether | 240.9 | 205.9 | 240.9 | 203.9 |
| 38 | Endosulfan-lacton | 355.8 | 320.9 | 355.8 | 277.1 |
| 39 | Endosulfan-sulfate | 387 | 253 | 387 | 217 |
| 40 | Endrin | 263 | 193 | 263 | 228 |
| 41 | Ethoprophos | 158 | 97.1 | 158 | 80.9 |
| 42 | Etoxazole | 359.1 | 330.2 | 359.1 | 339.1 |
| 43 | Fenarimol | 251 | 139 | 219 | 107 |

| | | T1 | | T2 | |
|---|---|---|---|---|---|
| | | Precursor ion (*m/z*) | Product ion (*m/z*) | Precursor ion (*m/z*) | Product ion (*m/z*) |
| 44 | Fenazaquin | 160 | 145.2 | 145 | 91.1 |
| 45 | Fenbuconazole | 198 | 102 | 198 | 128 |
| 46 | Fenitrothion | 277.1 | 109 | 277.1 | 124.9 |
| 47 | Fenpropathrin | 181 | 152 | 181 | 126 |
| 48 | Fenvalerate-I | 167 | 125 | 225 | 119 |
| 49 | Fenvalerate-II | 167 | 125 | 225 | 119 |
| 50 | Fludioxonil | 248 | 127 | 248 | 154 |
| 51 | Fluvalinate-tau-I | 208.9 | 77 | 250 | 199 |
| 52 | Fluvalinate-tau-II | 208.9 | 77 | 250 | 199 |
| 53 | Folpet | 147 | 103.1 | 147 | 76 |
| 54 | HCH-alpha | 219 | 183 | 219 | 145 |
| 55 | HCH-beta | 219 | 183 | 219 | 145 |
| 56 | HCH-delta | 219 | 183 | 219 | 145 |
| 57 | Hexachlorobenzene | 283.9 | 248.8 | 283.9 | 213.9 |
| 58 | Hexaconazole | 213.9 | 159 | 213.9 | 172 |
| 59 | Iprodione | 314 | 245.1 | 314 | 271 |
| 60 | Iprovalicarb-I | 158 | 116 | 134 | 93 |
| 61 | Iprovalicarb-II | 158 | 116 | 134 | 93 |
| 62 | Lenacil | 153 | 136 | 153 | 82 |
| 63 | Lindane | 218.8 | 183 | 180.9 | 145 |
| 64 | MCPA methyl ester | 214.1 | 141 | 214.1 | 155 |
| 65 | Methamidophos | 141 | 95 | 141 | 79 |
| 66 | Molinate | 126.1 | 98.1 | 126.1 | 81.3 |
| 67 | Omethoate | 110 | 79 | 156 | 110 |
| 68 | opDDD | 235 | 165.1 | 237 | 165 |
| 69 | opDDE | 246 | 176.1 | 248 | 176 |
| 70 | opDDT | 235 | 165 | 237 | 165 |
| 71 | o-Phenylphenol | 170 | 169 | 169 | 141 |
| 72 | Oxychlordane | 184.8 | 121 | 184.8 | 149 |
| 73 | PCB 101 | 326 | 291 | 326 | 256 |
| 74 | PCB 118 | 325.9 | 256.1 | 325.9 | 254.1 |
| 75 | PCB 138 | 359.9 | 324.8 | 359.9 | 289.8 |
| 76 | PCB 153 | 359.9 | 290 | 359.9 | 325 |
| 77 | PCB 180 | 393.9 | 358.9 | 393.9 | 323.8 |
| 78 | PCB 28 | 257.9 | 186 | 257.9 | 223 |
| 79 | PCB 52 | 292 | 257 | 292 | 222.1 |
| 80 | Pendimethalin | 252.1 | 162.1 | 252.1 | 161.2 |
| 81 | Permethrin-I | 183 | 168 | 183 | 153 |
| 82 | Permethrin-II | 183 | 77.1 | 183 | 115.2 |
| 83 | Phosmet | 160 | 77.1 | 160 | 133.1 |
| 84 | Pirimicarb | 238 | 166 | 166 | 96 |
| 85 | Pirimicarb desmethyl | 151.9 | 96.1 | 151.9 | 55 |
| 86 | ppDDD | 235 | 165 | 237 | 165 |
| 87 | ppDDE | 246 | 176.1 | 248 | 176 |
| 88 | ppDDT | 235 | 165 | 237 | 165 |
| 89 | Prochloraz | 180 | 69 | 180 | 138 |

| | | T1 | | T2 | |
|---|---|---|---|---|---|
| | | Precursor ion (*m/z*) | Product ion (*m/z*) | Precursor ion (*m/z*) | Product ion (*m/z*) |
| 90 | Profenofos | 208 | 63.1 | 208 | 98.1 |
| 91 | Propargite | 135.1 | 107.1 | 135.1 | 77 |
| 92 | Propiconazole-I | 259 | 69 | 259 | 173 |
| 93 | Propiconazole-II | 259 | 69 | 259 | 173 |
| 94 | Prothiofos | 267 | 239 | 309 | 239 |
| 95 | Pyridaben | 147 | 117 | 147 | 132 |
| 96 | Resmethrin | 171.1 | 143.1 | 171.1 | 128.1 |
| 97 | Spirodiclofen | 99 | 71 | 312 | 109 |
| 98 | Tefluthrin | 177 | 127 | 177 | 137 |
| 99 | Tetraconazole | 336 | 218 | 336 | 335 |
| 100 | Tetramethrin-I | 164 | 77 | 164 | 107 |
| 101 | Tetramethrin-II | 164 | 77 | 164 | 107 |
| 102 | Tolclofos-methyl | 265 | 250 | 265 | 93 |
| 103 | Triadimefon | 208 | 181 | 208 | 127 |
| 104 | Triadimenol-I | 168 | 70 | 112 | 83 |
| 105 | Triadimenol-II | 168 | 70 | 112 | 83 |
| 106 | Trifluralin | 305.9 | 264.1 | 263.9 | 160.1 |
| 107 | Aclonifen | 264 | 194 | 264 | 212.3 |
| 108 | Alachlor | 160.1 | 130 | 160.1 | 131.1 |
| 109 | Azaconazole | 217 | 173.1 | 217 | 145 |
| 110 | Azamethiophos | 215 | 171 | 215 | 128 |
| 111 | Azinphos-ethyl | 160.1 | 77.1 | 160.1 | 102 |
| 112 | Bromophos-ethyl | 358.7 | 303 | 358.7 | 331 |
| 113 | Bromophos-methyl | 331 | 316 | 331 | 286 |
| 114 | Chlorbufam | 223 | 171 | 223 | 164.1 |
| 115 | Chlorobenzilate | 139 | 111 | 139 | 75 |
| 116 | Chlozolinate | 330.8 | 259.1 | 188.1 | 147.1 |
| 117 | Coumaphos | 362.9 | 109.2 | 362.9 | 226.2 |
| 118 | Cyanofenphos | 168.9 | 141 | 168.9 | 77 |
| 119 | Cyanophos | 243 | 109 | 243 | 116 |
| 120 | Demeton-S-me-sulfone | 169 | 125 | 169 | 109 |
| 121 | Dichlobenil | 171 | 136 | 171 | 100 |
| 122 | Dichlofluanid | 224 | 123 | 167.3 | 124.1 |
| 123 | Dichlorvos | 109 | 79 | 185 | 93 |
| 124 | Dicloran | 205.9 | 176 | 175.9 | 148 |
| 125 | Dimoxystrobin | 115.9 | 89.1 | 204.9 | 116.2 |
| 126 | EPN | 157 | 77.1 | 157 | 110 |
| 127 | Etridazole | 210.9 | 182.9 | 182.9 | 139.9 |
| 128 | Fenamidone | 268.2 | 180.1 | 268.2 | 195.2 |
| 129 | Fenchlorphos | 285 | 270 | 285 | 93 |
| 130 | Flucythrinate-I | 199 | 107 | 199 | 157 |
| 131 | Flucythrinate-II | 199 | 107 | 199 | 157 |
| 132 | Flurtamone | 333.2 | 120.1 | 199 | 157.3 |
| 133 | Flusilazole | 233 | 165 | 233 | 152 |
| 134 | Fonofos | 246 | 109.1 | 246 | 81.1 |
| 135 | Formothion | 198 | 170 | 224 | 155 |

| | | T1 | | T2 | |
|---|---|---|---|---|---|
| | | Precursor ion (*m/z*) | Product ion (*m/z*) | Precursor ion (*m/z*) | Product ion (*m/z*) |
| 136 | Furalaxyl | 242 | 95 | 95 | 67 |
| 137 | Heptachlor | 272 | 237 | 274 | 239 |
| 138 | Heptachlor endo-epoxide,trans | 135 | 99 | 183 | 155 |
| 139 | Heptachlor exo-epoxide,cis | 352.9 | 262.9 | 352.9 | 281.9 |
| 140 | Iodofenphos | 376.9 | 361.9 | 376.9 | 93 |
| 141 | Isazophos | 161.1 | 119 | 161.1 | 146 |
| 142 | Isocarbofos | 136 | 108.3 | 120 | 92.3 |
| 143 | Isodrin | 192.9 | 123 | 192.9 | 157.1 |
| 144 | Isofenphos | 213 | 121.1 | 255 | 121 |
| 145 | Isofenphos-methyl | 199 | 121 | 199 | 167 |
| 146 | Isofenphos-oxon | 228.9 | 201 | 228.9 | 121 |
| 147 | Mecarbam | 131 | 42 | 159 | 73.9 |
| 148 | Methacrifos | 207.9 | 180 | 207.9 | 93 |
| 149 | Methoxychlor | 227 | 169 | 227 | 141.1 |
| 150 | Metribuzin | 198.1 | 82.1 | 198.1 | 89 |
| 151 | Mevinphos | 127 | 109 | 192 | 127 |
| 152 | Mirex | 272 | 237 | 272 | 235 |
| 153 | Nitrofen | 283 | 253 | 283 | 202 |
| 154 | Nonachlor-trans | 408.8 | 299.8 | 408.8 | 301.8 |
| 155 | Nuarimol | 314 | 139 | 235 | 139 |
| 156 | Oxadixyl | 163 | 132 | 233 | 118 |
| 157 | Paraoxon methyl | 230 | 200 | 230 | 136 |
| 158 | Parathion-ethyl | 291 | 109 | 291.1 | 81 |
| 159 | Parathion-methyl | 263 | 109.1 | 263 | 79.1 |
| 160 | Pentachloroaniline | 265 | 194 | 265 | 158 |
| 161 | Phenthoate | 274 | 125 | 274 | 121 |
| 162 | Phorate | 260 | 75 | 260 | 231 |
| 163 | Phosalone | 182 | 138 | 182 | 111 |
| 164 | Phosphamidon-I | 127 | 109 | 264 | 127 |
| 165 | Phosphamidon-II | 127 | 109 | 264 | 127 |
| 166 | ppDDE | 246 | 176.1 | 248 | 176 |
| 167 | Procymidone | 283 | 96 | 283 | 255 |
| 168 | Propachlor | 176 | 120 | 120.1 | 77.1 |
| 169 | Propanil | 161 | 126 | 163 | 162 |
| 170 | Propetamphos | 138 | 63.9 | 194.3 | 166 |
| 171 | Propham | 179 | 93 | 179 | 137 |
| 172 | Pyrifenox-I | 262 | 91.2 | 262 | 227 |
| 173 | Pyrifenox-II | 262 | 91.2 | 262 | 227 |
| 174 | Quintozene | 294.7 | 236.7 | 294.7 | 264.8 |
| 175 | Silthiofam | 252.1 | 75 | 252.1 | 197 |
| 176 | Tecnazene | 261 | 203 | 215 | 179 |
| 177 | Tetradifon | 354 | 159 | 356 | 159 |
| 178 | Tolyfluanid | 238 | 137 | 181 | 138 |
| 179 | Triflumizole | 206 | 179.2 | 287 | 218 |
| 180 | Vinclozolin | 212 | 172 | 212 | 145 |

| | | T1 | | T2 | |
|---|---|---|---|---|---|
| | | Precursor ion (*m/z*) | Product ion (*m/z*) | Precursor ion (*m/z*) | Product ion (*m/z*) |
| | | **LC Compounds** | | | |
| 181 | 1-Naphthylacetamide | 186 | 141 | 186 | 115 |
| 182 | Acephate | 184 | 143 | 184 | 95 |
| 183 | Acetamiprid | 223.1 | 126 | 223.1 | 90 |
| 184 | Atrazine | 216.1 | 174.1 | 216.1 | 68.1 |
| 185 | Atrazine-desethyl | 188.1 | 146 | 188.1 | 104 |
| 186 | Atrazine-desisopropyl | 174.1 | 96.1 | 174.1 | 132 |
| 187 | Azoxystrobin | 404.1 | 372.1 | 404.1 | 344.1 |
| 188 | BAC10 | 276.5 | 91 | 276.5 | 184 |
| 189 | BAC12 | 304 | 91 | 304 | 212 |
| 190 | BAC14 | 332 | 240 | 332 | 91 |
| 191 | BAC16 | 360.5 | 268 | 360.5 | 91 |
| 192 | Benalaxyl | 326.2 | 148.1 | 326.2 | 294 |
| 193 | Bendiocarb | 224.1 | 109 | 224.1 | 167.1 |
| 194 | Boscalid | 343 | 307.1 | 343 | 271 |
| 195 | Bromacil | 261 | 205 | 261 | 187.9 |
| 196 | Bupirimate | 317.2 | 166.1 | 317.2 | 108 |
| 197 | Buprofezin | 306.2 | 201.1 | 306.2 | 116.1 |
| 198 | Carbaryl | 202.1 | 145.1 | 202.1 | 127 |
| 199 | Carbendazim | 192.1 | 160.1 | 192.1 | 132.1 |
| 200 | Carbofuran | 222.1 | 165.1 | 222.1 | 123 |
| 201 | Carbofuran 3 Hydroxy | 238.1 | 181.1 | 238.1 | 163.1 |
| 202 | Carbosulfan | 381 | 118 | 381 | 160 |
| 203 | Chlorantraniliprole | 484 | 285.8 | 484 | 452.8 |
| 204 | Chlorfenvinphos | 359 | 155.1 | 359 | 127 |
| 205 | Chlorpyriphos | 350 | 96.9 | 350 | 197.8 |
| 206 | Clofentezine | 303 | 138 | 303 | 102 |
| 207 | Cyazofamid | 325.1 | 108 | 325.1 | 261 |
| 208 | Cymoxanil | 199.1 | 111 | 199.1 | 128.1 |
| 209 | Cyproconazole I | 292.1 | 125 | 292.1 | 70 |
| 210 | Cyproconazole II | 292.1 | 125 | 292.1 | 70 |
| 211 | Cyprodinil | 226.1 | 93 | 226.1 | 77 |
| 212 | DDAC | 326 | 186 | 326 | 44 |
| 213 | Diethofencarb | 268.2 | 124 | 268.2 | 226.1 |
| 214 | Difenoconazole | 406 | 250.7 | 406 | 187.8 |
| 215 | Dimethomorph I | 388.1 | 301.1 | 388.1 | 165 |
| 216 | Dimethomorph II | 388.1 | 301.1 | 388.1 | 165 |
| 217 | Dodine | 228 | 57 | 228 | 60 |
| 218 | Emamectin benzoate | 886.5 | 158 | 886.5 | 126 |
| 219 | Epoxyconazole | 330.1 | 121 | 330.1 | 100.9 |
| 220 | Ethiofencarb Sulfoxide | 242 | 107 | 242 | 185 |
| 221 | Ethion | 385 | 198.9 | 385 | 142.9 |
| 222 | Ethirimol | 210.2 | 97.9 | 210.2 | 139.7 |
| 223 | Etofenprox | 394.2 | 177.1 | 394.2 | 107 |
| 224 | Famoxadone | 392.2 | 238.1 | 392.2 | 331.1 |

| | | T1 | | T2 | |
|---|---|---|---|---|---|
| | | Precursor ion (*m/z*) | Product ion (*m/z*) | Precursor ion (*m/z*) | Product ion (*m/z*) |
| 225 | Fenhexamid | 302.1 | 97.1 | 302.1 | 55.1 |
| 226 | Fenoxycarb | 302.1 | 88 | 302.1 | 116.1 |
| 227 | Fenpropidin | 274.3 | 147.1 | 275.3 | 148.1 |
| 228 | Fenpropimorph | 304.3 | 147.1 | 304.3 | 130 |
| | | | | 304.3 | 117 |
| | | | | 304.3 | 69 |
| 229 | Fenpyroximate | 422.2 | 366 | 422.2 | 135 |
| 230 | Fensulfothion | 309.1 | 173 | 309.1 | 252.9 |
| 231 | Fenthion | 279.1 | 246.8 | 279.1 | 168.9 |
| 232 | Fenthion Sulfone | 310.8 | 124.9 | 310.8 | 109 |
| 233 | Fenthion Sulfoxide | 294.9 | 279.8 | 294.9 | 109 |
| 234 | Flonicamid | 230.1 | 203 | 230.1 | 174 |
| 235 | Fludioxonil | 266.1 | 229 | 266.1 | 158 |
| 236 | Flufenoxuron | 489.2 | 158.1 | 489.2 | 141.1 |
| 237 | Fluopicolide | 383 | 173 | 383 | 145 |
| 238 | Fluopyram | 396.4 | 207.4 | 396.4 | 172.7 |
| 239 | Fluquinconazole | 376 | 307 | 376 | 349 |
| 240 | Flutolanil | 324.1 | 242.1 | 324.1 | 262.1 |
| 241 | Flutriafol | 302.1 | 70 | 302.1 | 123 |
| 242 | Fluxapyroxad | 382.2 | 362.2 | 382.2 | 342.2 |
| 243 | Fosthiazate | 284.1 | 227.8 | 284.1 | 103.8 |
| 244 | Hexythiazox | 353.1 | 228 | 353.1 | 168.1 |
| 245 | Imazalil | 297.1 | 159 | 297.1 | 201 |
| 246 | Imidacloprid | 256.1 | 209.1 | 256.1 | 175 |
| 247 | Indoxacarb | 528.1 | 203 | 528.1 | 150 |
| 248 | Isoprothiolane | 291.1 | 231.1 | 291.1 | 189 |
| 249 | Kresoxim-methyl | 314.1 | 206 | 314.1 | 267 |
| 250 | Linuron | 249 | 160 | 249 | 182 |
| 251 | Malaoxon | 315.1 | 126.8 | 315.1 | 99 |
| 252 | Malathion | 331 | 127 | 331 | 285 |
| 253 | Mandipropamid | 412 | 328 | 412 | 356 |
| 254 | Mepanipyrim | 224.1 | 106 | 224.1 | 77 |
| 255 | Metalaxyl | 280.2 | 220.1 | 280.2 | 160 |
| | | | | 280.2 | 192.1 |
| 256 | Metamitron | 203.1 | 175.1 | 203.1 | 104.1 |
| 257 | Methamidophos | 142 | 94 | 142 | 125 |
| 258 | Methidathion | 303 | 145 | 303 | 85 |
| 259 | Methiocarb | 226.1 | 169.1 | 226.1 | 121.1 |
| 260 | Methiocarb Sulfone | 275 | 201 | 275 | 122 |
| 261 | Methiocarb Sulfoxide | 242.3 | 153 | 242.3 | 170 |
| 262 | Methomyl | 163 | 88 | 163 | 106 |
| 263 | Methoxyfenozide | 369.2 | 149.1 | 369.2 | 133 |
| 264 | Metrafenone | 409 | 209 | 409 | 227 |
| 265 | Molinate | 188.1 | 126.1 | 188.1 | 98 |
| 266 | Monocrotophos | 224.1 | 127 | 224.1 | 193 |
| 267 | Myclobutanil | 289.1 | 70 | 289.1 | 125 |

| | | T1 | | T2 | |
|---|---|---|---|---|---|
| | | Precursor ion (*m/z*) | Product ion (*m/z*) | Precursor ion (*m/z*) | Product ion (*m/z*) |
| 268 | Oxamyl | 237 | 72 | 237 | 90 |
| 269 | Paclobutrazol | 294 | 70 | 294 | 125 |
| 270 | Penconazole | 284.1 | 70 | 284.1 | 159 |
| 271 | Pencycuron | 329.1 | 125 | 329.1 | 218 |
| 272 | Phenmedipham | 301.1 | 168.1 | 301.1 | 136.1 |
| 273 | Piperonyl butoxide | 356.2 | 177.1 | 356.2 | 119 |
| 274 | Pirimiphos-ethyl | 334.1 | 198 | 334.1 | 182.1 |
| 275 | Pirimiphos-methyl | 306.1 | 164.2 | 306.1 | 108 |
| 276 | Propyzamide | 256 | 190 | 256 | 173 |
| 277 | Proquinazid | 373.1 | 331 | 373.1 | 289 |
| 278 | Prosulfocarb | 252.1 | 91.1 | 252.1 | 128.1 |
| 279 | Prothioconazole desthio | 311.7 | 69.7 | 311.7 | 125 |
| 280 | Pymetrozine | 218.1 | 105 | 218.1 | 79 |
| 281 | Pyraclostrobin | 388.1 | 194.1 | 388.1 | 163 |
| 282 | Pyrethrins | 373.2 | 161.1 | 373.2 | 133.1 |
| 283 | Pyridaben | 365.1 | 147 | 365.1 | 309.1 |
| 284 | Pyridalyl | 492 | 111 | 492 | 164 |
| 285 | Pyrimethanil | 200.1 | 107 | 200.1 | 82 |
| 286 | Pyriproxifen | 322.1 | 96 | 322.1 | 185 |
| 287 | Quinoxyfen | 308 | 162 | 308 | 197 |
| 288 | Spinosyn A | 732.5 | 142.1 | 732.5 | 98 |
| 289 | Spinosyn D | 746.5 | 142.1 | 746.5 | 98 |
| 290 | Spirodiclofen | 411.1 | 71.2 | 411.1 | 313 |
| 291 | Spiromesifen | 371 | 273 | 371 | 255 |
| 292 | Spirotetramat | 374 | 330 | 374 | 302 |
| 293 | Spiroxamine | 298.3 | 144.1 | 298.3 | 100.1 |
| 294 | Tebuconazole | 308.2 | 70 | 308.2 | 125 |
| 295 | Tebufenozide | 353.2 | 133.1 | 353.2 | 297.1 |
| 296 | Tebufenpyrad | 334.2 | 145.1 | 334.2 | 147 |
| | | | | 334.2 | 132.1 |
| 297 | Terbuthylazine | 230.1 | 174.1 | 230.1 | 132 |
| 298 | Thiabendazole | 202 | 175 | 202 | 131.1 |
| 299 | Thiacloprid | 253 | 126 | 253 | 90 |
| 300 | Thiamethoxam | 292 | 211 | 292 | 181 |
| 301 | Thiodicarb | 355 | 87.9 | 355 | 107.9 |
| 302 | Thiophanate-Ethyl | 370.8 | 324.8 | 370.8 | 150.9 |
| 303 | Thiophanate-Methyl | 343.1 | 151 | 343.1 | 311 |
| 304 | Triazophos | 314.1 | 162.1 | 314.1 | 119 |
| 305 | Trichlorfon | 256.9 | 221 | 256.9 | 109 |
| 306 | Tricyclazole | 190.1 | 162.7 | 190.1 | 135.7 |
| 307 | Trifloxystrobin | 409.1 | 186.1 | 409.1 | 145 |
| 308 | Zoxamide | 336 | 187 | 336 | 159 |
| 309 | 2,4,5-T | 254.9 | 196.8 | 254.9 | 160.9 |
| 310 | 2,4-D | 221 | 163 | 219 | 161 |
| 311 | 2,4-DB | 247 | 161 | 249 | 163 |
| 312 | Bentazone | 239.1 | 132.1 | 239.1 | 197 |

| | | T1 | | T2 | |
|---|---|---|---|---|---|
| | | Precursor ion (*m/z*) | Product ion (*m/z*) | Precursor ion (*m/z*) | Product ion (*m/z*) |
| 313 | Bixafen | 412 | 279.4 | 412 | 130.7 |
| 314 | Bromoxynil | 273.9 | 78.9 | 275.9 | 80.9 |
| 315 | Chlorfluazuron | 538 | 517.9 | 538 | 354.9 |
| 316 | Clethodim | 358.1 | 238.1 | 358.1 | 268 |
| 317 | Clothianidin | 247.9 | 164.8 | 247.9 | 211.7 |
| 318 | Cyclanilide | 272 | 159.9 | 272 | 228 |
| 319 | Cycloxydim | 324.2 | 235.9 | 324.2 | 133.8 |
| 320 | Dichlorprop | 233 | 161 | 235 | 163 |
| 321 | Diflubenzuron | 309 | 288.9 | 309 | 155.9 |
| 322 | Dinoseb | 239.1 | 134 | 239.1 | 193 |
| 323 | Dinoterb | 238.9 | 176 | 238.9 | 45.9 |
| 324 | DNOC | 197 | 137 | 197 | 108.8 |
| 325 | Endosulfan sulfate | 420.8 | 96.8 | 420.8 | 79.9 |
| 326 | Fenoprop (2,4,5 TP) | 266.9 | 194.8 | 266.9 | 158.9 |
| 327 | Fipronil | 435 | 329.9 | 435 | 249.9 |
| 328 | Fipronil desulfynil | 387 | 351 | 387 | 282 |
| 329 | Fipronil sulfide | 419 | 383 | 419 | 262 |
| 330 | Fipronil sulfone | 451 | 415 | 451 | 282 |
| 331 | Fluazifop | 326.1 | 253.8 | 326.1 | 108.1 |
| 332 | Fluazinam | 462.9 | 415.9 | 462.9 | 398 |
| 333 | Flubendiamide | 681 | 253.9 | 681 | 271.7 |
| 334 | Haloxyfop | 360 | 287.9 | 360 | 227.4 |
| 335 | Hexaflumuron | 459 | 439 | 459 | 175 |
| 336 | Ioxynil | 369.8 | 127 | 369.8 | 214.9 |
| 337 | MCPA | 199 | 141 | 201 | 143 |
| 338 | MCPB | 227 | 141 | 229 | 143 |
| 339 | Mecoprop | 213 | 141 | 215 | 143 |
| 340 | Quizalofop | 343.1 | 270.9 | 343.1 | 242.9 |
| 341 | Sulfentrazone | 385 | 307.1 | 385 | 198.9 |
| 342 | Teflubenzuron | 379 | 338.9 | 379 | 359 |
| 343 | TFNA | 190 | 146 | 190 | 99 |
| | | | | 190 | 69 |
| 344 | TFNG | 247 | 146 | 247 | 163 |
| | | | | 247 | 183 |
| 345 | Triclopyr | 255.9 | 198 | 253.9 | 196 |
| 346 | Triflumuron | 357 | 154 | 357 | 175.9 |

**Table – S5.** Recovery data of 346 pesticides analysed in GC-MS/MS and UHPLC-MS. The data was calculated from two sets of repeatability experiments performed with 7 repetitions at 10µg/kg and with 6 repetitions at 100µg/kg. In this study, the lower concentration level corresponds to the limit of quantitation (LOQ). ME corresponds to the percentage of observed matrix effect for honey and pollen in 180 pesticides detected in GC-MS/MS. Standards were matrix matched with Ethyl-acetate and blank samples (honey and pollen) where matrix matched with standard solutions. An effect from -20 to 20% was considered as non-significative. Non applicable (NA) was used for compounds where ME was not determined.

| No. | Compound | LOQ (µg/kg) | At 10 µg/kg | | | At 100 µg/kg | | | ME (%) | |
|---|---|---|---|---|---|---|---|---|---|---|
| | | | %Recovery (AVG) | SD | %RSD | %Recovery (AVG) | SD | %RSD | Honey | Pollen |
| | **GC Compounds** | | | | | | | | | |
| 1 | 1,4-Dimethylnapthalene | 10.0 | 64.8 | 6.0 | 9.3 | 56.2 | 10.0 | 17.7 | 7.2 | 18.0 |
| 2 | 4,4-Dichlorobenzophenone | 10.0 | 105.9 | 3.8 | 3.6 | 100.5 | 7.4 | 7.4 | -29.8 | 10.9 |
| 3 | Acephate | 10.0 | 90.3 | 8.6 | 9.5 | 79.1 | 6.3 | 7.9 | 248.1 | 231.6 |
| 4 | Aclonifen | 10.0 | 84.6 | 32.3 | 38.2 | 74.2 | 8.5 | 11.4 | -13.8 | 47.7 |
| 5 | Acrinathrin | 10.0 | 110.5 | 19.3 | 17.5 | 91.2 | 6.0 | 6.6 | -25.9 | 35.9 |
| 6 | Alachlor | 51.0 | 91.0 | 10.8 | 11.8 | 94.5 | 7.7 | 8.2 | -6.0 | 25.4 |
| 7 | Aldrin | 10.0 | 84.8 | 5.6 | 6.6 | 88.7 | 6.0 | 6.7 | -22.8 | 14.6 |
| 8 | Anthraquinone | 10.0 | 93.7 | 3.6 | 3.8 | 93.7 | 6.0 | 6.4 | -10.5 | 22.0 |
| 9 | Azaconazole | 10.0 | 94.3 | 4.6 | 4.9 | 93.0 | 5.1 | 5.5 | -10.3 | 20.2 |
| 10 | Azamethiophos | 10.0 | 108.9 | 36.5 | 33.5 | 100.7 | 9.1 | 9.1 | 384.2 | 1059.6 |
| 11 | Azinphos-ethyl | 50.0 | 103.6 | 17.1 | 16.5 | 95.3 | 6.5 | 6.9 | -3.7 | 93.0 |
| 12 | Azinphos-methyl | 10.0 | 111.3 | 18.2 | 16.3 | 93.8 | 6.0 | 6.4 | 63.3 | 558.2 |
| 13 | Azoxystrobin | 10.0 | 44.3 | 91.2 | 205.8 | 94.6 | 5.4 | 5.7 | -29.7 | 32.9 |
| 14 | Bifenthrin | 10.0 | 95.4 | 5.4 | 5.7 | 95.3 | 7.1 | 7.4 | -16.8 | 27.3 |
| 15 | Binapacryl | 10.0 | 0.0 | 0.0 | 0.0 | 76.5 | 24.5 | 32.1 | -22.0 | -651.3 |
| 16 | Biphenyl | 10.0 | 62.8 | 5.5 | 8.7 | 51.7 | 9.1 | 17.6 | 1.8 | 15.4 |
| 17 | Bitertanol-I | 10.0 | 98.1 | 20.7 | 21.1 | 98.6 | 7.2 | 7.3 | -13.3 | -6.4 |
| 18 | Bitertanol-II | 10.0 | 98.1 | 20.7 | 21.1 | 99.9 | 12.1 | 12.2 | 58.1 | -74.8 |
| 19 | Boscalid | 10.0 | 141.5 | 36.2 | 25.6 | 100.7 | 9.6 | 9.5 | -28.5 | 13.2 |
| 20 | Bromophos-ethyl | 10.0 | 95.1 | 11.3 | 11.9 | 92.6 | 6.7 | 7.2 | -16.2 | 32.3 |
| 21 | Bromophos-methyl | 10.0 | 91.3 | 9.2 | 10.1 | 90.8 | 6.2 | 6.9 | -10.1 | 75.8 |

| No. | Compound | LOQ (µg/kg) | At 10 µg/kg | | | At 100 µg/kg | | | ME (%) | |
|---|---|---|---|---|---|---|---|---|---|---|
| | | | %Recovery (AVG) | SD | %RSD | %Recovery (AVG) | SD | %RSD | Honey | Pollen |
| 22 | Bromopropylate | 10.0 | 90.7 | 4.2 | 4.6 | 91.6 | 6.4 | 7.0 | -20.8 | 11.5 |
| 23 | Captafol | 10.0 | 0.0 | 0.0 | 0.0 | 176.9 | 100.5 | 56.8 | 0.0 | -122.0 |
| 24 | Captan | 10.0 | 0.0 | 0.0 | 0.0 | 119.7 | 14.3 | 11.9 | 10.8 | 150.2 |
| 25 | Chlorbufam | 10.0 | 100.6 | 14.3 | 14.2 | 91.9 | 9.0 | 9.8 | 38.6 | 46.1 |
| 26 | Chlordane-cis | 10.0 | 74.1 | 9.4 | 12.7 | 94.2 | 6.6 | 7.0 | -26.0 | 15.5 |
| 27 | Chlordane-trans | 20.0 | 88.5 | 15.8 | 17.9 | 94.2 | 7.3 | 7.7 | -28.8 | 40.0 |
| 28 | Chlorfenapyr | 10.0 | 85.4 | 12.6 | 14.8 | 97.3 | 4.1 | 4.2 | -17.7 | 45.7 |
| 29 | Chlorobenzilate | 10.0 | 96.8 | 7.8 | 8.1 | 96.7 | 5.9 | 6.1 | -11.7 | 29.2 |
| 30 | Chlorothalonil | 10.0 | 123.3 | 14.3 | 11.6 | 87.7 | 6.8 | 7.8 | 228.2 | 170.7 |
| 31 | Chlorpropham | 10.0 | 93.1 | 6.9 | 7.4 | 88.8 | 7.4 | 8.3 | 57.2 | 31.1 |
| 32 | Chlorpyrifos methyl | 10.0 | 97.5 | 10.3 | 10.6 | 87.9 | 7.3 | 8.3 | -3.2 | 58.8 |
| 33 | Chlorthal-dimethyl | 10.0 | 99.0 | 8.1 | 8.2 | 93.5 | 6.7 | 7.2 | -24.5 | 17.2 |
| 34 | Chlozolinate | 50.0 | 78.4 | 17.8 | 22.7 | 93.8 | 5.6 | 6.0 | -17.1 | 7.1 |
| 35 | Coumaphos | 10.0 | 166.4 | 39.0 | 23.5 | 99.5 | 11.4 | 11.5 | -37.3 | 185.4 |
| 36 | Cyanofenphos | 10.0 | 88.3 | 5.4 | 6.1 | 92.8 | 5.9 | 6.3 | -15.0 | 49.2 |
| 37 | Cyanophos | 10.0 | 94.4 | 6.9 | 7.3 | 90.9 | 7.5 | 8.3 | 47.0 | 68.9 |
| 38 | Cyfluthrin | 10.0 | 164.5 | 104.5 | 63.5 | 95.2 | 10.0 | 10.5 | -7.6 | -78.2 |
| 39 | Cyhalothrin-lambda | 10.0 | 102.1 | 30.9 | 30.3 | 91.4 | 6.6 | 7.2 | -11.5 | 37.2 |
| 40 | Cypermethrin | 10.0 | 0.0 | 0.0 | 0.0 | 95.9 | 7.7 | 8.1 | -13.6 | -29.6 |
| 41 | Cyproconazole | 10.0 | 105.1 | 6.5 | 6.2 | 90.9 | 4.9 | 5.4 | -9.3 | 30.3 |
| 42 | Deltamethrin | 10.0 | 142.2 | 112.8 | 79.3 | 87.9 | 6.8 | 7.7 | -19.3 | -4.6 |
| 43 | Demeton-S-me-sulfone | 10.0 | 113.3 | 9.7 | 8.5 | 98.8 | 8.8 | 8.9 | 268.9 | 74.7 |
| 44 | Diazinon | 10.0 | 87.6 | 10.8 | 12.3 | 93.1 | 7.4 | 8.0 | 13.1 | 28.8 |
| 45 | Dichlobenil | 10.0 | 70.0 | 5.6 | 8.1 | 54.6 | 12.9 | 23.7 | -0.6 | 10.6 |
| 46 | Dichlofluanid | 10.0 | 118.9 | 14.9 | 12.5 | 99.8 | 9.4 | 9.4 | -3.3 | 72.1 |
| 47 | Dichlorvos | 10.0 | 87.3 | 3.8 | 4.4 | 61.9 | 23.0 | 37.1 | 4.3 | 23.2 |
| 48 | Dicloran | 10.0 | 96.9 | 3.8 | 4.0 | 84.3 | 6.9 | 8.2 | 58.7 | 36.5 |
| 49 | Dicofol | 10.0 | 0.0 | 0.0 | 0.0 | 231.7 | 237.7 | 102.6 | 16481.6 | 750.7 |

| No. | Compound | LOQ (µg/kg) | At 10 µg/kg | | | At 100 µg/kg | | | ME (%) | |
|---|---|---|---|---|---|---|---|---|---|---|
| | | | %Recovery (AVG) | SD | %RSD | %Recovery (AVG) | SD | %RSD | Honey | Pollen |
| 50 | Dieldrin | 10.0 | 82.3 | 11.6 | 14.1 | 91.7 | 4.2 | 4.5 | -18.2 | -3.0 |
| 51 | Dimethoate | 10.0 | 97.0 | 7.2 | 7.4 | 93.9 | 8.3 | 8.8 | 93.9 | 122.5 |
| 52 | Dimoxystrobin | 10.0 | 88.4 | 6.4 | 7.2 | 96.7 | 6.3 | 6.5 | -13.2 | 32.6 |
| 53 | Diphenylamine | 10.0 | 134.0 | 8.5 | 6.3 | 89.1 | 8.0 | 9.0 | 17.7 | 18.9 |
| 54 | Endosulfan-alpha | 10.0 | 75.0 | 13.6 | 18.1 | 91.1 | 7.9 | 8.7 | -22.0 | 24.1 |
| 55 | Endosulfan-beta | 10.0 | 76.8 | 21.2 | 27.7 | 95.9 | 4.4 | 4.6 | -24.3 | 22.6 |
| 56 | Endosulfan-ether | 10.0 | 85.6 | 8.5 | 9.9 | 87.3 | 9.2 | 10.6 | -14.9 | -2.9 |
| 57 | Endosulfan-lacton | 10.0 | 102.0 | 24.5 | 24.0 | 98.3 | 5.1 | 5.2 | -22.1 | 7.7 |
| 58 | Endosulfan-sulfate | 10.0 | 114.6 | 58.6 | 51.1 | 87.4 | 4.8 | 5.4 | -17.5 | 152.4 |
| 59 | Endrin | 10.0 | 92.2 | 29.5 | 32.0 | 91.8 | 7.5 | 8.1 | -13.6 | 43.3 |
| 60 | EPN | 10.0 | 102.9 | 14.2 | 13.8 | 79.4 | 12.2 | 15.4 | -17.2 | 42.2 |
| 61 | Ethoprophos | 10.0 | 96.6 | 6.8 | 7.0 | 86.7 | 8.8 | 10.1 | 22.5 | 28.3 |
| 62 | Etoxazole | 10.0 | 0.0 | 0.0 | 0.0 | 95.8 | 6.3 | 6.6 | -27.4 | -8.0 |
| 63 | Etridazole | 10.0 | 124.9 | 21.4 | 17.2 | 60.4 | 13.6 | 22.5 | 11.7 | 260.1 |
| 64 | Fenamidone | 10.0 | 131.9 | 38.0 | 28.8 | 106.8 | 5.9 | 5.6 | -19.4 | 29.9 |
| 65 | Fenarimol | 10.0 | 98.9 | 17.7 | 17.9 | 96.4 | 7.9 | 8.2 | -19.8 | 23.2 |
| 66 | Fenazaquin | 10.0 | 93.6 | 10.0 | 10.6 | 95.5 | 6.7 | 7.0 | -9.0 | 33.4 |
| 67 | Fenbuconazole | 10.0 | 106.0 | 18.3 | 17.3 | 105.2 | 11.6 | 11.1 | -18.8 | 28.5 |
| 68 | Fenchlorphos | 10.0 | 98.8 | 7.0 | 7.1 | 90.1 | 5.7 | 6.4 | 6.4 | 66.3 |
| 69 | Fenitrothion | 10.0 | 112.4 | 4.6 | 4.1 | 86.5 | 7.4 | 8.5 | 19.0 | 110.7 |
| 70 | Fenpropathrin | 10.0 | 103.4 | 23.4 | 22.6 | 96.2 | 7.2 | 7.5 | -11.8 | -34.7 |
| 71 | Fenvalerate-I | 10.0 | 146.3 | 136.6 | 93.4 | 94.4 | 5.8 | 6.1 | -13.3 | 26.1 |
| 72 | Fenvalerate-II | 10.0 | 0.0 | 0.0 | 0.0 | 94.1 | 6.9 | 7.4 | -16.3 | 33.5 |
| 73 | Flucythrinate-I | 5.0 | 124.6 | 86.6 | 69.5 | 97.5 | 9.3 | 9.5 | -27.8 | 4.7 |
| 74 | Flucythrinate-II | 5.0 | 136.3 | 71.1 | 52.2 | 94.2 | 7.1 | 7.5 | -28.9 | -19.2 |
| 75 | Fludioxonil | 5.0 | 91.2 | 11.5 | 12.6 | 99.1 | 6.2 | 6.3 | 13.6 | 35.2 |
| 76 | Flurtamone | 5.0 | 142.6 | 12.7 | 8.9 | 90.9 | 15.1 | 16.6 | -24.6 | 46.3 |
| 77 | Flusilazole | 5.0 | 89.8 | 7.0 | 7.8 | 96.3 | 6.2 | 6.5 | -21.6 | 18.5 |

| No. | Compound | LOQ (µg/kg) | At 10 µg/kg | | | At 100 µg/kg | | | ME (%) | |
|---|---|---|---|---|---|---|---|---|---|---|
| | | | %Recovery (AVG) | SD | %RSD | %Recovery (AVG) | SD | %RSD | Honey | Pollen |
| 78 | Fluvalinate-tau-I | 5.0 | 20.4 | 33.9 | 166.2 | 112.2 | 9.3 | 8.3 | -24.6 | 12.7 |
| 79 | Fluvalinate-tau-II | 5.0 | 65.7 | 59.1 | 89.9 | 110.2 | 6.9 | 6.3 | -25.2 | 5.6 |
| 80 | Folpet | 10.0 | 0.0 | 0.0 | 0.0 | 124.7 | 13.9 | 11.1 | 0.5 | 561.7 |
| 81 | Fonofos | 10.0 | 94.9 | 8.1 | 8.5 | 88.3 | 9.4 | 10.7 | 23.4 | 24.7 |
| 82 | Formothion | 10.0 | 137.1 | 19.9 | 14.5 | 93.3 | 6.5 | 6.9 | 27.3 | 124.9 |
| 83 | Furalaxyl | 10.0 | 99.2 | 10.8 | 10.9 | 94.8 | 5.8 | 6.2 | -15.9 | 25.1 |
| 84 | HCH-alpha | 10.0 | 109.2 | 6.1 | 5.6 | 81.3 | 7.9 | 9.8 | 17.5 | 21.9 |
| 85 | HCH-beta | 10.0 | 126.6 | 12.0 | 9.5 | 93.3 | 6.4 | 6.8 | 8.4 | 38.6 |
| 86 | HCH-delta | 10.0 | 85.8 | 10.5 | 12.2 | 89.4 | 5.1 | 5.7 | 21.1 | 47.8 |
| 87 | Heptachlor | 10.0 | 114.6 | 10.4 | 9.0 | 87.4 | 8.5 | 9.7 | -3.2 | 61.1 |
| 88 | Heptachlor endo-epoxide,trans | 10.0 | 88.7 | 9.4 | 10.6 | 97.9 | 7.5 | 7.7 | -11.7 | 27.7 |
| 89 | Heptachlor exo-epoxide,cis | 10.0 | 105.5 | 9.6 | 9.1 | 89.9 | 5.6 | 6.3 | -14.8 | 10.9 |
| 90 | Hexachlorobenzene | 10.0 | 78.6 | 6.2 | 7.9 | 76.3 | 7.1 | 9.3 | 8.0 | 13.1 |
| 91 | Hexaconazole | 10.0 | 86.7 | 13.9 | 16.1 | 99.1 | 4.5 | 4.5 | -1.5 | -32.6 |
| 92 | Iodofenphos | 10.0 | 104.9 | 7.8 | 7.5 | 89.5 | 5.3 | 5.9 | 4.5 | 115.9 |
| 93 | Iprodione | 10.0 | 83.7 | 15.4 | 18.4 | 92.7 | 6.0 | 6.5 | -13.3 | 1.9 |
| 94 | Iprovalicarb-I | 10.0 | 132.8 | 10.1 | 7.6 | 98.9 | 7.1 | 7.2 | 11.3 | 43.9 |
| 95 | Iprovalicarb-II | 10.0 | 92.1 | 14.5 | 15.8 | 95.3 | 5.7 | 6.0 | 6.4 | 43.7 |
| 96 | Isazophos | 100.0 | 85.8 | 7.6 | 8.9 | 88.8 | 8.0 | 9.0 | -1.3 | 30.3 |
| 97 | Isocarbofos | 10.0 | 114.5 | 16.4 | 14.3 | 99.0 | 5.1 | 5.2 | 0.3 | 46.4 |
| 98 | Isodrin | 10.0 | 85.7 | 3.2 | 3.7 | 92.2 | 6.8 | 7.4 | -15.4 | 23.2 |
| 99 | Isofenphos | 10.0 | 101.7 | 5.5 | 5.4 | 99.5 | 5.8 | 5.8 | -2.6 | 31.0 |
| 100 | Isofenphos-methyl | 20.0 | 101.7 | 4.5 | 4.4 | 98.9 | 6.9 | 6.9 | -5.1 | 37.5 |
| 101 | Isofenphos-oxon | 20.0 | 100.4 | 6.8 | 6.8 | 103.0 | 11.3 | 10.9 | -2.3 | 70.9 |
| 102 | Lenacil | 10.0 | 90.3 | 8.4 | 9.3 | 92.9 | 4.9 | 5.3 | 0.6 | 13.2 |
| 103 | Lindane | 10.0 | 111.6 | 9.2 | 8.3 | 87.1 | 7.9 | 9.1 | 8.0 | 35.3 |
| 104 | MCPA methyl ester | 10.0 | 83.4 | 6.6 | 7.9 | 80.0 | 8.9 | 11.1 | 14.2 | 23.6 |
| 105 | Mecarbam | 10.0 | 89.2 | 16.4 | 18.3 | 97.7 | 6.6 | 6.8 | -5.9 | 39.3 |

| No. | Compound | LOQ (µg/kg) | At 10 µg/kg | | | At 100 µg/kg | | | ME (%) | |
|---|---|---|---|---|---|---|---|---|---|---|
| | | | %Recovery (AVG) | SD | %RSD | %Recovery (AVG) | SD | %RSD | Honey | Pollen |
| 106 | Methacrifos | 10.0 | 76.1 | 5.6 | 7.4 | 72.2 | 10.8 | 15.0 | 8.9 | 28.0 |
| 107 | Methamidophos | 10.0 | 76.3 | 4.6 | 6.0 | 69.8 | 28.3 | 40.6 | 59.2 | 123.4 |
| 108 | Methoxychlor | 10.0 | 144.0 | 160.0 | 111.1 | 138.8 | 43.8 | 31.5 | -22.6 | 166.4 |
| 109 | Metribuzin | 10.0 | 91.4 | 2.1 | 2.3 | 85.2 | 5.2 | 6.1 | -0.6 | -13.9 |
| 110 | Mevinphos | 10.0 | 93.2 | 8.2 | 8.8 | 86.3 | 9.1 | 10.6 | 26.5 | 60.5 |
| 111 | Mirex | 10.0 | 102.5 | 12.1 | 11.8 | 96.5 | 6.5 | 6.7 | -23.2 | 23.9 |
| 112 | Molinate | 10.0 | 82.6 | 8.7 | 10.5 | 70.0 | 10.2 | 14.6 | 7.0 | 17.6 |
| 113 | Nitrofen | 10.0 | 82.3 | 27.7 | 33.7 | 76.4 | 6.5 | 8.5 | -7.3 | 54.0 |
| 114 | Nonachlor-trans | 10.0 | 106.2 | 19.7 | 18.5 | 93.3 | 6.1 | 6.6 | -10.9 | 5.2 |
| 115 | Nuarimol | 10.0 | 72.1 | 14.3 | 19.8 | 91.2 | 6.8 | 7.5 | -19.9 | 16.6 |
| 116 | Omethoate | 10.0 | 91.2 | 5.5 | 6.1 | 90.4 | 6.1 | 6.8 | 310.0 | 293.6 |
| 117 | opDDD | 10.0 | 97.2 | 3.7 | 3.8 | 90.7 | 6.8 | 7.5 | -9.0 | 10.1 |
| 118 | opDDE | 10.0 | 89.3 | 6.3 | 7.0 | 93.7 | 6.0 | 6.4 | -14.2 | 16.5 |
| 119 | opDDT | 10.0 | 101.3 | 4.3 | 4.2 | 93.6 | 5.9 | 6.3 | -7.1 | 38.9 |
| 120 | o-Phenylphenol | 10.0 | 139.2 | 5.7 | 4.1 | 91.9 | 9.4 | 10.3 | 39.1 | 34.5 |
| 121 | Oxadixyl | 10.0 | 99.5 | 8.7 | 8.8 | 97.8 | 4.4 | 4.5 | -13.0 | 31.3 |
| 122 | Oxychlordane | 10.0 | 94.0 | 5.2 | 5.5 | 94.0 | 7.3 | 7.7 | -17.1 | 32.2 |
| 123 | Paraoxon methyl | 10.0 | 108.9 | 18.5 | 17.0 | 89.3 | 6.6 | 7.4 | 151.9 | 302.8 |
| 124 | Parathion-ethyl | 10.0 | 117.0 | 6.5 | 5.5 | 88.0 | 6.7 | 7.6 | -4.6 | 61.3 |
| 125 | Parathion-methyl | 10.0 | 117.3 | 4.7 | 4.0 | 81.4 | 6.4 | 7.9 | 23.3 | 118.6 |
| 126 | PCB 101 | 10.0 | 82.7 | 12.5 | 15.1 | 89.9 | 6.0 | 6.6 | -25.0 | 8.4 |
| 127 | PCB 118 | 10.0 | 87.6 | 15.9 | 18.1 | 89.1 | 7.5 | 8.4 | -22.8 | 6.6 |
| 128 | PCB 138 | 10.0 | 85.2 | 17.6 | 20.7 | 87.7 | 6.6 | 7.5 | -21.7 | 21.5 |
| 129 | PCB 153 | 10.0 | 78.3 | 17.2 | 22.0 | 89.9 | 6.5 | 7.2 | -23.1 | -31.0 |
| 130 | PCB 180 | 10.0 | 109.3 | 19.8 | 18.1 | 87.5 | 4.9 | 5.6 | -30.0 | 15.1 |
| 131 | PCB 28 | 10.0 | 91.8 | 6.1 | 6.7 | 87.6 | 7.8 | 8.9 | -6.4 | 18.2 |
| 132 | PCB 52 | 10.0 | 87.1 | 6.2 | 7.1 | 90.4 | 6.0 | 6.6 | -7.7 | 16.2 |
| 133 | Pendimethalin | 10.0 | 101.9 | 14.9 | 14.6 | 80.6 | 5.9 | 7.3 | -11.1 | 39.6 |

| No. | Compound | LOQ (µg/kg) | At 10 µg/kg | | | At 100 µg/kg | | | ME (%) | |
|---|---|---|---|---|---|---|---|---|---|---|
| | | | %Recovery (AVG) | SD | %RSD | %Recovery (AVG) | SD | %RSD | Honey | Pollen |
| 134 | Pentachloroaniline | 10.0 | 87.3 | 8.0 | 9.1 | 88.3 | 6.7 | 7.6 | -2.9 | 19.9 |
| 135 | Permethrin-I | 10.0 | 362.9 | 294.6 | 81.2 | 104.3 | 13.4 | 12.8 | -7.1 | 15.1 |
| 136 | Permethrin-II | 10.0 | 69.8 | 54.1 | 77.5 | 100.5 | 12.5 | 12.4 | -16.6 | 13.3 |
| 137 | Phenthoate | 10.0 | 101.0 | 6.4 | 6.4 | 94.5 | 7.0 | 7.4 | -5.6 | 50.6 |
| 138 | Phorate | 10.0 | 247.9 | 25.6 | 10.3 | 113.0 | 18.0 | 15.9 | 60.0 | 142.8 |
| 139 | Phosalone | 10.0 | 86.3 | 18.2 | 21.1 | 94.3 | 5.5 | 5.9 | 1.6 | 175.2 |
| 140 | Phosmet | 10.0 | 103.4 | 6.6 | 6.4 | 92.2 | 5.2 | 5.6 | 27.3 | 248.3 |
| 141 | Phosphamidon-I | 10.0 | 95.2 | 21.5 | 22.6 | 102.7 | 9.1 | 8.9 | 63.3 | 188.5 |
| 142 | Phosphamidon-II | 10.0 | 101.4 | 8.1 | 8.0 | 95.4 | 7.5 | 7.9 | 24.2 | 129.6 |
| 143 | Pirimicarb | 10.0 | 80.9 | 11.6 | 14.4 | 97.6 | 7.3 | 7.5 | -0.8 | 42.7 |
| 144 | Pirimicarb desmethyl | 10.0 | 93.9 | 8.6 | 9.2 | 92.1 | 4.0 | 4.4 | -29.4 | -65.0 |
| 145 | ppDDD | 10.0 | 101.3 | 4.3 | 4.2 | 93.6 | 5.9 | 6.3 | -7.1 | 38.9 |
| 146 | ppDDE | 10.0 | 88.6 | 5.8 | 6.5 | 92.5 | 5.1 | 5.5 | -11.3 | 16.1 |
| 147 | ppDDE | 10.0 | 88.6 | 5.8 | 6.5 | 92.5 | 5.1 | 5.5 | -6.7 | 16.1 |
| 148 | ppDDT | 10.0 | 168.4 | 0.1 | 0.1 | 115.7 | 30.3 | 26.1 | -13.3 | 243.4 |
| 149 | Prochloraz | 10.0 | 24.2 | 54.1 | 223.3 | 88.7 | 6.9 | 7.8 | -27.1 | 6.5 |
| 150 | Procymidone | 10.0 | 92.6 | 21.6 | 23.3 | 91.7 | 5.2 | 5.6 | -5.0 | 18.9 |
| 151 | Profenofos | 10.0 | 98.9 | 2.8 | 2.8 | 98.0 | 5.5 | 5.6 | 1.7 | 106.4 |
| 152 | Propachlor | 10.0 | 91.1 | 13.1 | 14.3 | 90.4 | 8.0 | 8.9 | 17.2 | 30.4 |
| 153 | Propanil | 10.0 | 95.7 | 17.6 | 18.4 | 94.6 | 6.7 | 7.1 | 6.5 | 36.9 |
| 154 | Propargite | 10.0 | 76.3 | 15.8 | 20.7 | 121.5 | 43.9 | 36.1 | -44.2 | 29.4 |
| 155 | Propetamphos | 10.0 | 97.3 | 6.4 | 6.6 | 94.8 | 7.1 | 7.5 | 21.6 | 33.1 |
| 156 | Propham | 10.0 | 85.1 | 6.5 | 7.7 | 76.5 | 10.9 | 14.3 | 13.3 | 20.7 |
| 157 | Propiconazole-I | 10.0 | 114.8 | 23.2 | 20.2 | 94.0 | 6.7 | 7.1 | -11.3 | 26.0 |
| 158 | Propiconazole-II | 10.0 | 100.7 | 14.0 | 13.9 | 88.2 | 6.5 | 7.3 | -4.9 | 17.8 |
| 159 | Prothiofos | 10.0 | 120.6 | 6.8 | 5.6 | 93.2 | 5.1 | 5.5 | 0.1 | 31.1 |
| 160 | Pyridaben | 10.0 | 79.3 | 16.7 | 21.1 | 97.0 | 10.7 | 11.0 | -15.3 | 21.1 |
| 161 | Pyrifenox-I | 10.0 | 90.8 | 10.1 | 11.1 | 99.2 | 6.3 | 6.3 | 1.0 | 31.0 |

| No. | Compound | LOQ (µg/kg) | At 10 µg/kg | | | At 100 µg/kg | | | ME (%) | |
|---|---|---|---|---|---|---|---|---|---|---|
| | | | %Recovery (AVG) | SD | %RSD | %Recovery (AVG) | SD | %RSD | Honey | Pollen |
| 162 | Pyrifenox-II | 10.0 | 80.0 | 40.2 | 50.3 | 95.4 | 9.6 | 10.0 | -1.6 | 27.6 |
| 163 | Quintozene | 10.0 | 92.4 | 7.7 | 8.3 | 76.9 | 7.6 | 9.9 | 24.6 | 30.5 |
| 164 | Resmethrin | 10.0 | 88.1 | 7.7 | 8.7 | 92.4 | 6.5 | 7.0 | -11.7 | 25.7 |
| 165 | Silthiofam | 10.0 | 93.3 | 9.5 | 10.2 | 97.7 | 9.0 | 9.2 | -6.2 | 32.0 |
| 166 | Spirodiclofen | 10.0 | 0.0 | 0.0 | 0.0 | 108.6 | 12.8 | 11.8 | 23.4 | -115.0 |
| 167 | Tecnazene | 10.0 | 84.3 | 7.2 | 8.5 | 72.3 | 11.2 | 15.5 | 11.6 | 20.4 |
| 168 | Tefluthrin | 10.0 | 92.7 | 7.2 | 7.7 | 89.3 | 7.5 | 8.3 | -2.8 | 23.3 |
| 169 | Tetraconazole | 10.0 | 98.4 | 6.8 | 6.9 | 99.5 | 7.8 | 7.8 | -21.4 | 26.9 |
| 170 | Tetradifon | 10.0 | 101.7 | 19.7 | 19.4 | 94.8 | 9.2 | 9.7 | -22.5 | 24.8 |
| 171 | Tetramethrin-I | 10.0 | 99.7 | 11.2 | 11.2 | 92.4 | 6.3 | 6.9 | -3.0 | 7.4 |
| 172 | Tetramethrin-II | 10.0 | 93.2 | 10.4 | 11.1 | 94.4 | 6.3 | 6.7 | -9.0 | 35.9 |
| 173 | Tolclofos-methyl | 10.0 | 96.5 | 7.0 | 7.2 | 91.5 | 6.8 | 7.4 | -1.8 | 32.3 |
| 174 | Tolyfluanid | 10.0 | 116.0 | 9.3 | 8.0 | 97.6 | 9.4 | 9.7 | -4.9 | 67.3 |
| 175 | Triadimefon | 10.0 | 101.7 | 5.3 | 5.2 | 99.2 | 7.4 | 7.5 | -13.0 | 32.5 |
| 176 | Triadimenol-I | 10.0 | 110.9 | 23.5 | 21.1 | 97.4 | 5.8 | 5.9 | -3.5 | 34.6 |
| 177 | Triadimenol-II | 10.0 | 110.9 | 23.5 | 21.1 | 106.0 | 7.5 | 7.0 | 12.7 | 623.1 |
| 178 | Triflumizole | 10.0 | 90.7 | 7.1 | 7.8 | 86.3 | 10.5 | 12.2 | -11.2 | -71.8 |
| 179 | Trifluralin | 10.0 | 105.5 | 7.1 | 6.7 | 84.2 | 8.9 | 10.6 | 20.9 | 38.4 |
| 180 | Vinclozolin | 10.0 | 98.8 | 17.3 | 17.5 | 92.6 | 7.7 | 8.4 | 4.0 | 25.0 |
| | **LC Compounds** | | | | | | | | | |
| 181 | 1-Naphthylacetamide | 10.0 | 106.9 | 3.1 | 2.9 | 105.5 | 3.1 | 3.0 | NA | NA |
| 182 | Acephate | 10.0 | 79.8 | 8.5 | 10.7 | 77.2 | 6.4 | 8.2 | NA | NA |
| 183 | Acetamiprid | 10.0 | 98.0 | 6.6 | 6.7 | 116.3 | 5.0 | 4.3 | NA | NA |
| 184 | Atrazine | 10.0 | 117.8 | 3.9 | 3.3 | 110.5 | 6.6 | 6.0 | NA | NA |
| 185 | Atrazine-desethyl | 10.0 | 114.4 | 4.1 | 3.6 | 107.5 | 6.4 | 6.0 | NA | NA |
| 186 | Atrazine-desisopropyl | 10.0 | 54.3 | 8.0 | 14.7 | 99.5 | 5.6 | 5.7 | NA | NA |
| 187 | Azoxystrobin | 10.0 | 110.8 | 4.8 | 4.4 | 107.3 | 4.6 | 4.2 | NA | NA |

| No. | Compound | LOQ (µg/kg) | At 10 µg/kg | | | At 100 µg/kg | | | ME (%) | |
|---|---|---|---|---|---|---|---|---|---|---|
| | | | %Recovery (AVG) | SD | %RSD | %Recovery (AVG) | SD | %RSD | Honey | Pollen |
| 188 | BAC10 | 10.0 | 76.4 | 6.9 | 9.0 | 49.4 | 4.4 | 8.9 | NA | NA |
| 189 | BAC12 | 10.0 | 153.3 | 104.4 | 68.1 | 34.5 | 19.1 | 55.3 | NA | NA |
| 190 | BAC14 | 10.0 | 107.0 | 64.5 | 60.3 | 39.4 | 15.8 | 40.1 | NA | NA |
| 191 | BAC16 | 10.0 | 95.1 | 21.5 | 22.7 | 40.5 | 6.4 | 15.8 | NA | NA |
| 192 | Benalaxyl | 10.0 | 109.1 | 4.6 | 4.2 | 106.8 | 8.5 | 7.9 | NA | NA |
| 193 | Bendiocarb | 10.0 | 97.6 | 6.6 | 6.8 | 107.6 | 2.7 | 2.5 | NA | NA |
| 194 | Boscalid | 10.0 | 141.2 | 8.9 | 6.3 | 108.0 | 7.7 | 7.1 | NA | NA |
| 195 | Bromacil | 10.0 | 95.2 | 4.4 | 4.6 | 108.1 | 6.1 | 5.7 | NA | NA |
| 196 | Bupirimate | 10.0 | 110.8 | 3.4 | 3.1 | 114.6 | 4.9 | 4.3 | NA | NA |
| 197 | Buprofezin | 10.0 | 106.0 | 13.2 | 12.5 | 102.6 | 6.4 | 6.3 | NA | NA |
| 198 | Carbaryl | 10.0 | 108.8 | 7.4 | 6.8 | 107.7 | 5.6 | 5.2 | NA | NA |
| 199 | Carbendazim | 10.0 | 126.7 | 6.1 | 4.8 | 113.8 | 5.9 | 5.1 | NA | NA |
| 200 | Carbofuran | 10.0 | 126.1 | 5.9 | 4.7 | 108.3 | 4.6 | 4.3 | NA | NA |
| 201 | Carbofuran 3 Hydroxy | 10.0 | 120.8 | 7.2 | 5.9 | 104.9 | 3.4 | 3.2 | NA | NA |
| 202 | Carbosulfan | 10.0 | 97.0 | 3.6 | 3.7 | 97.1 | 4.0 | 4.1 | NA | NA |
| 203 | Chlorantraniliprole | 10.0 | 109.4 | 5.6 | 5.1 | 111.7 | 6.2 | 5.5 | NA | NA |
| 204 | Chlorfenvinphos | 10.0 | 121.6 | 2.1 | 1.7 | 113.0 | 8.3 | 7.3 | NA | NA |
| 205 | Chlorpyriphos | 10.0 | 257.2 | 5.8 | 2.3 | 116.8 | 5.1 | 4.4 | NA | NA |
| 206 | Clofentezine | 10.0 | 118.3 | 4.6 | 3.9 | 104.3 | 3.8 | 3.7 | NA | NA |
| 207 | Cyazofamid | 10.0 | 136.3 | 5.8 | 4.2 | 138.6 | 5.9 | 4.2 | NA | NA |
| 208 | Cymoxanil | 10.0 | 113.2 | 30.6 | 27.0 | 106.9 | 5.7 | 5.3 | NA | NA |
| 209 | Cyproconazole I | 10.0 | 193.5 | 8.0 | 4.1 | 108.2 | 6.5 | 6.0 | NA | NA |
| 210 | Cyproconazole II | 10.0 | 47.2 | 13.3 | 28.3 | 105.4 | 10.0 | 9.5 | NA | NA |
| 211 | Cyprodinil | 10.0 | 116.1 | 4.3 | 3.7 | 109.9 | 7.1 | 6.4 | NA | NA |
| 212 | DDAC | 10.0 | 31.2 | 27.9 | 89.4 | 49.5 | 8.8 | 17.8 | NA | NA |
| 213 | Diethofencarb | 10.0 | 97.4 | 7.8 | 8.0 | 104.6 | 10.2 | 9.8 | NA | NA |
| 214 | Difenoconazole | 10.0 | 117.0 | 8.8 | 7.5 | 134.4 | 14.6 | 10.9 | NA | NA |
| 215 | Dimethomorph I | 10.0 | 101.1 | 3.2 | 3.1 | 126.3 | 6.5 | 5.1 | NA | NA |

| No. | Compound | LOQ (µg/kg) | At 10 µg/kg | | | At 100 µg/kg | | | ME (%) | |
|---|---|---|---|---|---|---|---|---|---|---|
| | | | %Recovery (AVG) | SD | %RSD | %Recovery (AVG) | SD | %RSD | Honey | Pollen |
| 216 | Dimethomorph II | 10.0 | 111.2 | 3.8 | 3.4 | 104.6 | 6.1 | 5.8 | NA | NA |
| 217 | Dodine | 10.0 | 0.0 | 0.0 | 0.0 | 0.0 | 0.0 | 0.0 | NA | NA |
| 218 | Emamectin benzoate | 10.0 | 27.9 | 4.4 | 15.6 | 68.2 | 7.4 | 10.9 | NA | NA |
| 219 | Epoxyconazole | 10.0 | 113.1 | 3.4 | 3.0 | 109.2 | 6.0 | 5.5 | NA | NA |
| 220 | Ethiofencarb Sulfoxide | 49.0 | 45.4 | 4.0 | 8.8 | 98.1 | 4.4 | 4.5 | NA | NA |
| 221 | Ethion | 10.0 | 107.3 | 4.2 | 3.9 | 103.5 | 7.1 | 6.9 | NA | NA |
| 222 | Ethirimol | 10.0 | 102.9 | 5.6 | 5.4 | 100.9 | 8.3 | 8.2 | NA | NA |
| 223 | Etofenprox | 10.0 | 71.3 | 5.1 | 7.2 | 57.0 | 8.9 | 15.6 | NA | NA |
| 224 | Famoxadone | 10.0 | 109.0 | 8.5 | 7.8 | 109.1 | 5.7 | 5.2 | NA | NA |
| 225 | Fenhexamid | 10.0 | 116.7 | 9.5 | 8.1 | 114.4 | 3.5 | 3.0 | NA | NA |
| 226 | Fenoxycarb | 10.0 | 91.5 | 4.1 | 4.5 | 109.2 | 6.6 | 6.0 | NA | NA |
| 227 | Fenpropidin | 10.0 | 71.9 | 4.3 | 6.0 | 97.2 | 3.5 | 3.6 | NA | NA |
| 228 | Fenpropimorph | 10.0 | 96.9 | 4.7 | 4.9 | 104.1 | 2.6 | 2.5 | NA | NA |
| 229 | Fenpyroximate | 10.0 | 108.1 | 4.9 | 4.6 | 105.9 | 6.6 | 6.2 | NA | NA |
| 230 | Fensulfothion | 10.0 | 98.9 | 7.9 | 8.0 | 108.7 | 10.7 | 9.9 | NA | NA |
| 231 | Fenthion | 10.0 | 100.2 | 15.7 | 15.7 | 94.9 | 15.0 | 15.8 | NA | NA |
| 232 | Fenthion Sulfone | 10.0 | 106.0 | 8.6 | 8.2 | 107.3 | 9.1 | 8.5 | NA | NA |
| 233 | Fenthion Sulfoxide | 10.0 | 130.4 | 5.0 | 3.9 | 117.7 | 10.8 | 9.2 | NA | NA |
| 234 | Flonicamid | 10.0 | 97.2 | 4.4 | 4.5 | 93.7 | 6.0 | 6.5 | NA | NA |
| 235 | Fludioxonil | 10.0 | 114.5 | 12.7 | 11.1 | 104.6 | 4.5 | 4.3 | NA | NA |
| 236 | Flufenoxuron | 10.0 | 123.8 | 4.9 | 4.0 | 105.4 | 5.6 | 5.4 | NA | NA |
| 237 | Fluopicolide | 10.0 | 97.3 | 6.1 | 6.2 | 110.4 | 1.6 | 1.4 | NA | NA |
| 238 | Fluopyram | 20.0 | 114.4 | 32.1 | 28.1 | 120.4 | 16.0 | 13.3 | NA | NA |
| 239 | Fluquinconazole | 10.0 | 149.0 | 6.3 | 4.3 | 123.0 | 7.6 | 6.2 | NA | NA |
| 240 | Flutolanil | 10.0 | 114.3 | 4.5 | 3.9 | 117.2 | 7.1 | 6.1 | NA | NA |
| 241 | Flutriafol | 10.0 | 122.5 | 2.2 | 1.8 | 109.6 | 4.8 | 4.4 | NA | NA |
| 242 | Fluxapyroxad | 10.0 | 107.1 | 3.0 | 2.8 | 112.8 | 4.7 | 4.2 | NA | NA |
| 243 | Fosthiazate | 10.0 | 115.9 | 6.3 | 5.4 | 102.4 | 4.7 | 4.6 | NA | NA |

| No. | Compound | LOQ (μg/kg) | At 10 μg/kg | | | At 100 μg/kg | | | ME (%) | |
|---|---|---|---|---|---|---|---|---|---|---|
| | | | %Recovery (AVG) | SD | %RSD | %Recovery (AVG) | SD | %RSD | Honey | Pollen |
| 244 | Hexythiazox | 10.0 | 115.5 | 3.6 | 3.1 | 99.6 | 2.6 | 2.7 | NA | NA |
| 245 | Imazalil | 10.0 | 111.1 | 6.2 | 5.6 | 107.7 | 8.1 | 7.5 | NA | NA |
| 246 | Imidacloprid | 10.0 | 45.1 | 3.6 | 8.1 | 104.0 | 4.1 | 3.9 | NA | NA |
| 247 | Indoxacarb | 10.0 | 111.0 | 8.4 | 7.6 | 104.3 | 10.6 | 10.2 | NA | NA |
| 248 | Isoprothiolane | 10.0 | 114.2 | 3.9 | 3.4 | 110.5 | 4.1 | 3.7 | NA | NA |
| 249 | Kresoxim-methyl | 10.0 | 116.2 | 8.8 | 7.5 | 101.8 | 5.9 | 5.8 | NA | NA |
| 250 | Linuron | 10.0 | 101.6 | 6.3 | 6.3 | 106.1 | 6.7 | 6.3 | NA | NA |
| 251 | Malaoxon | 10.0 | 111.2 | 5.4 | 4.9 | 108.6 | 5.4 | 5.0 | NA | NA |
| 252 | Malathion | 10.0 | 107.6 | 4.0 | 3.7 | 109.9 | 5.4 | 4.9 | NA | NA |
| 253 | Mandipropamid | 10.0 | 115.6 | 8.0 | 7.0 | 113.0 | 7.0 | 6.2 | NA | NA |
| 254 | Mepanipyrim | 10.0 | 116.4 | 4.6 | 4.0 | 106.9 | 4.2 | 3.9 | NA | NA |
| 255 | Metalaxyl | 10.0 | 112.0 | 5.6 | 5.0 | 108.6 | 7.8 | 7.2 | NA | NA |
| 256 | Metamitron | 10.0 | 94.0 | 18.6 | 19.8 | 95.5 | 6.8 | 7.1 | NA | NA |
| 257 | Methamidophos | 10.0 | 82.8 | 2.1 | 2.6 | 64.1 | 5.2 | 8.1 | NA | NA |
| 258 | Methidathion | 10.0 | 111.3 | 10.5 | 9.4 | 110.8 | 8.4 | 7.5 | NA | NA |
| 259 | Methiocarb | 10.0 | 110.8 | 3.9 | 3.5 | 112.9 | 3.7 | 3.3 | NA | NA |
| 260 | Methiocarb Sulfone | 10.0 | 266.1 | 20.9 | 7.9 | 165.3 | 84.6 | 51.2 | NA | NA |
| 261 | Methiocarb Sulfoxide | 9.0 | 89.4 | 12.4 | 13.8 | 113.6 | 14.8 | 13.1 | NA | NA |
| 262 | Methomyl | 10.0 | 0.0 | 0.0 | 0.0 | 100.7 | 4.1 | 4.0 | NA | NA |
| 263 | Methoxyfenozide | 10.0 | 114.8 | 2.3 | 2.0 | 110.7 | 4.4 | 4.0 | NA | NA |
| 264 | Metrafenone | 10.0 | 102.3 | 4.6 | 4.5 | 106.8 | 4.2 | 3.9 | NA | NA |
| 265 | Molinate | 10.0 | 105.2 | 14.9 | 14.1 | 77.0 | 6.5 | 8.5 | NA | NA |
| 266 | Monocrotophos | 10.0 | 84.8 | 4.4 | 5.2 | 101.4 | 6.2 | 6.2 | NA | NA |
| 267 | Myclobutanil | 10.0 | 120.2 | 4.0 | 3.3 | 112.7 | 3.6 | 3.2 | NA | NA |
| 268 | Oxamyl | 10.0 | 108.0 | 3.8 | 3.5 | 100.7 | 2.8 | 2.8 | NA | NA |
| 269 | Paclobutrazol | 10.0 | 142.5 | 12.1 | 8.5 | 115.1 | 4.2 | 3.6 | NA | NA |
| 270 | Penconazole | 10.0 | 111.8 | 4.9 | 4.4 | 110.3 | 5.2 | 4.8 | NA | NA |
| 271 | Pencycuron | 10.0 | 121.6 | 3.7 | 3.1 | 111.5 | 6.8 | 6.1 | NA | NA |

| No. | Compound | LOQ (µg/kg) | At 10 µg/kg | | | At 100 µg/kg | | | ME (%) | |
|---|---|---|---|---|---|---|---|---|---|---|
| | | | %Recovery (AVG) | SD | %RSD | %Recovery (AVG) | SD | %RSD | Honey | Pollen |
| 272 | Phenmedipham | 10.0 | 122.9 | 6.1 | 4.9 | 108.1 | 4.7 | 4.3 | NA | NA |
| 273 | Piperonyl butoxide | 10.0 | 126.7 | 6.6 | 5.2 | 107.1 | 8.8 | 8.2 | NA | NA |
| 274 | Pirimiphos-ethyl | 10.0 | 119.7 | 3.9 | 3.2 | 102.0 | 3.2 | 3.1 | NA | NA |
| 275 | Pirimiphos-methyl | 10.0 | 117.1 | 4.5 | 3.8 | 106.3 | 7.1 | 6.7 | NA | NA |
| 276 | Propyzamide | 10.0 | 88.7 | 4.0 | 4.5 | 108.5 | 5.3 | 4.9 | NA | NA |
| 277 | Proquinazid | 10.0 | 111.4 | 4.1 | 3.7 | 110.1 | 6.1 | 5.6 | NA | NA |
| 278 | Prosulfocarb | 50.0 | 113.7 | 5.5 | 4.8 | 99.8 | 6.5 | 6.5 | NA | NA |
| 279 | Prothioconazole desthio | 10.0 | 103.8 | 9.0 | 8.6 | 110.3 | 7.0 | 6.4 | NA | NA |
| 280 | Pymetrozine | 10.0 | 38.4 | 1.2 | 3.2 | 41.2 | 7.5 | 18.2 | NA | NA |
| 281 | Pyraclostrobin | 10.0 | 119.1 | 5.1 | 4.3 | 108.8 | 5.6 | 5.1 | NA | NA |
| 282 | Pyrethrins | 49.0 | 101.8 | 3.5 | 3.4 | 103.2 | 5.9 | 5.7 | NA | NA |
| 283 | Pyridaben | 10.0 | 129.7 | 5.2 | 4.0 | 114.3 | 5.8 | 5.1 | NA | NA |
| 284 | Pyridalyl | 10.0 | 121.7 | 4.4 | 3.6 | 99.0 | 8.5 | 8.6 | NA | NA |
| 285 | Pyrimethanil | 10.0 | 138.3 | 12.6 | 9.1 | 112.5 | 4.4 | 3.9 | NA | NA |
| 286 | Pyriproxifen | 10.0 | 90.9 | 5.4 | 5.9 | 102.6 | 6.1 | 5.9 | NA | NA |
| 287 | Quinoxyfen | 10.0 | 108.0 | 10.8 | 10.0 | 107.7 | 4.9 | 4.6 | NA | NA |
| 288 | Spinosyn A | 10.0 | 62.7 | 7.1 | 11.4 | 92.9 | 3.7 | 4.0 | NA | NA |
| 289 | Spinosyn D | 10.0 | 67.3 | 4.6 | 6.8 | 90.3 | 5.4 | 6.0 | NA | NA |
| 290 | Spirodiclofen | 10.0 | 103.1 | 7.2 | 7.0 | 102.5 | 2.1 | 2.1 | NA | NA |
| 291 | Spiromesifen | 10.0 | 101.5 | 9.8 | 9.7 | 106.7 | 2.9 | 2.7 | NA | NA |
| 292 | Spirotetramat | 10.0 | 118.5 | 7.2 | 6.0 | 105.8 | 13.8 | 13.0 | NA | NA |
| 293 | Spiroxamine | 10.0 | 56.1 | 5.6 | 10.1 | 80.4 | 5.6 | 7.0 | NA | NA |
| 294 | Tebuconazole | 10.0 | 110.1 | 3.5 | 3.2 | 112.2 | 3.9 | 3.4 | NA | NA |
| 295 | Tebufenozide | 10.0 | 109.2 | 6.2 | 5.7 | 110.7 | 3.4 | 3.0 | NA | NA |
| 296 | Tebufenpyrad | 10.0 | 121.4 | 5.5 | 4.5 | 97.1 | 4.2 | 4.4 | NA | NA |
| 297 | Terbuthylazine | 10.0 | 106.5 | 3.3 | 3.1 | 109.1 | 2.4 | 2.2 | NA | NA |
| 298 | Thiabendazole | 10.0 | 232.0 | 20.8 | 9.0 | 109.1 | 33.3 | 30.5 | NA | NA |
| 299 | Thiacloprid | 10.0 | 93.9 | 5.2 | 5.5 | 111.3 | 10.2 | 9.1 | NA | NA |

| No. | Compound | LOQ (µg/kg) | At 10 µg/kg | | | At 100 µg/kg | | | ME (%) | |
|---|---|---|---|---|---|---|---|---|---|---|
| | | | %Recovery (AVG) | SD | %RSD | %Recovery (AVG) | SD | %RSD | Honey | Pollen |
| 300 | Thiamethoxam | 10.0 | 83.6 | 3.9 | 4.6 | 100.3 | 6.7 | 6.7 | NA | NA |
| 301 | Thiodicarb | 10.0 | 122.1 | 5.5 | 4.5 | 107.6 | 7.5 | 7.0 | NA | NA |
| 302 | Thiophanate-Ethyl | 10.0 | 238.9 | 317.7 | 133.0 | 75.1 | 60.5 | 80.6 | NA | NA |
| 303 | Thiophanate-Methyl | 10.0 | 132.2 | 13.1 | 9.9 | 15.9 | 1.1 | 7.1 | NA | NA |
| 304 | Triazophos | 10.0 | 222.2 | 4.4 | 2.0 | 116.1 | 5.1 | 4.4 | NA | NA |
| 305 | Trichlorfon | 20.0 | 85.1 | 12.4 | 14.5 | 107.8 | 12.4 | 11.5 | NA | NA |
| 306 | Tricyclazole | 10.0 | 115.5 | 3.2 | 2.7 | 103.0 | 8.6 | 8.4 | NA | NA |
| 307 | Trifloxystrobin | 10.0 | 111.3 | 4.7 | 4.2 | 106.8 | 5.3 | 4.9 | NA | NA |
| 308 | Zoxamide | 10.0 | 104.5 | 4.7 | 4.5 | 106.5 | 3.5 | 3.3 | NA | NA |
| 309 | 2,4,5-T | 10.0 | 111.3 | 10.3 | 9.3 | 36.9 | 23.7 | 64.3 | NA | NA |
| 310 | 2,4-D | 19.0 | 118.8 | 7.0 | 5.9 | 99.3 | 5.9 | 5.9 | NA | NA |
| 311 | 2,4-DB | 67.0 | 71.7 | 19.5 | 27.2 | 110.0 | 10.6 | 9.6 | NA | NA |
| 312 | Bentazone | 9.0 | 79.4 | 2.5 | 3.1 | 102.4 | 6.9 | 6.7 | NA | NA |
| 313 | Bixafen | 10.0 | 99.4 | 11.8 | 11.8 | 35.7 | 46.7 | 130.9 | NA | NA |
| 314 | Bromoxynil | 10.0 | 121.6 | 4.8 | 3.9 | 93.1 | 7.2 | 7.7 | NA | NA |
| 315 | Chlorfluazuron | 11.0 | 114.9 | 3.9 | 3.4 | 30.4 | 25.7 | 84.7 | NA | NA |
| 316 | Clethodim | 10.0 | 3.7 | 2.2 | 59.2 | 22.6 | 12.7 | 56.2 | NA | NA |
| 317 | Clothianidin | 10.0 | 98.3 | 4.4 | 4.5 | 92.5 | 9.6 | 10.4 | NA | NA |
| 318 | Cyclanilide | 10.0 | 120.1 | 7.6 | 6.4 | 87.2 | 15.7 | 17.9 | NA | NA |
| 319 | Cycloxydim | 101.0 | 0.0 | 0.0 | 0.0 | 0.0 | 0.0 | 0.0 | NA | NA |
| 320 | Dichlorprop | 11.0 | 100.2 | 7.0 | 7.0 | 111.7 | 13.0 | 11.6 | NA | NA |
| 321 | Diflubenzuron | 10.0 | 86.0 | 34.0 | 39.6 | 12.7 | 23.2 | 183.3 | NA | NA |
| 322 | Dinoseb | 10.0 | 91.2 | 5.2 | 5.7 | 82.6 | 10.5 | 12.7 | NA | NA |
| 323 | Dinoterb | 10.0 | 82.3 | 6.2 | 7.5 | 71.0 | 17.4 | 24.4 | NA | NA |
| 324 | DNOC | 10.0 | 109.5 | 42.9 | 39.2 | 80.7 | 8.0 | 9.8 | NA | NA |
| 325 | Endosulfan sulfate | 11.0 | 90.4 | 38.7 | 42.8 | 91.9 | 17.3 | 18.8 | NA | NA |
| 326 | Fenoprop (2,4,5 TP) | 10.0 | 111.4 | 10.5 | 9.4 | 64.8 | 32.7 | 50.5 | NA | NA |
| 327 | Fipronil | 10.0 | 109.1 | 4.4 | 4.1 | 97.5 | 12.2 | 12.5 | NA | NA |

| No. | Compound | LOQ (µg/kg) | At 10 µg/kg | | | At 100 µg/kg | | | ME (%) | |
|---|---|---|---|---|---|---|---|---|---|---|
| | | | %Recovery (AVG) | SD | %RSD | %Recovery (AVG) | SD | %RSD | Honey | Pollen |
| 328 | Fipronil desulfynil | 10.0 | 99.1 | 2.0 | 2.0 | 100.0 | 11.8 | 11.8 | NA | NA |
| 329 | Fipronil sulfide | 10.0 | 97.7 | 1.0 | 1.1 | 100.1 | 10.4 | 10.4 | NA | NA |
| 330 | Fipronil sulfone | 10.0 | 91.2 | 2.2 | 2.4 | 95.6 | 9.6 | 10.1 | NA | NA |
| 331 | Fluazifop | 10.0 | 115.9 | 8.9 | 7.7 | 20.8 | 27.2 | 130.9 | NA | NA |
| 332 | Fluazinam | 10.0 | 97.1 | 1.9 | 2.0 | 98.5 | 14.1 | 14.3 | NA | NA |
| 333 | Flubendiamide | 10.0 | 107.8 | 4.0 | 3.7 | 60.2 | 86.9 | 144.4 | NA | NA |
| 334 | Haloxyfop | 10.0 | 119.5 | 9.2 | 7.7 | 24.5 | 26.5 | 108.3 | NA | NA |
| 335 | Hexaflumuron | 10.0 | 108.0 | 2.7 | 2.5 | 105.0 | 14.7 | 14.0 | NA | NA |
| 336 | Ioxynil | 10.0 | 127.2 | 5.3 | 4.2 | 116.7 | 7.9 | 6.8 | NA | NA |
| 337 | MCPA | 10.0 | 268.4 | 36.7 | 13.7 | 98.0 | 9.0 | 9.2 | NA | NA |
| 338 | MCPB | 10.0 | 4.7 | 4.0 | 0.0 | 0.0 | 0.0 | 0.0 | NA | NA |
| 339 | Mecoprop | 10.0 | 105.0 | 6.6 | 6.3 | 94.0 | 7.9 | 8.4 | NA | NA |
| 340 | Quizalofop | 10.0 | 79.1 | 32.5 | 41.1 | 90.4 | 10.4 | 0.0 | NA | NA |
| 341 | Sulfentrazone | 9.0 | 73.5 | 2.8 | 3.8 | 69.0 | 20.9 | 30.3 | NA | NA |
| 342 | Teflubenzuron | 10.0 | 114.3 | 4.3 | 3.8 | 101.3 | 13.3 | 13.2 | NA | NA |
| 343 | TFNA | 10.0 | 452.2 | 757.1 | 167.4 | 71.4 | 11.5 | 16.1 | NA | NA |
| 344 | TFNG | 10.0 | 125.6 | 15.1 | 12.0 | 49.3 | 7.0 | 14.1 | NA | NA |
| 345 | Triclopyr | 10.0 | 126.5 | 26.1 | 20.7 | 103.7 | 14.3 | 13.8 | NA | NA |
| 346 | Triflumuron | 10.0 | 105.3 | 4.2 | 3.9 | 95.8 | 8.9 | 9.3 | NA | NA |

**Supplemental figure**

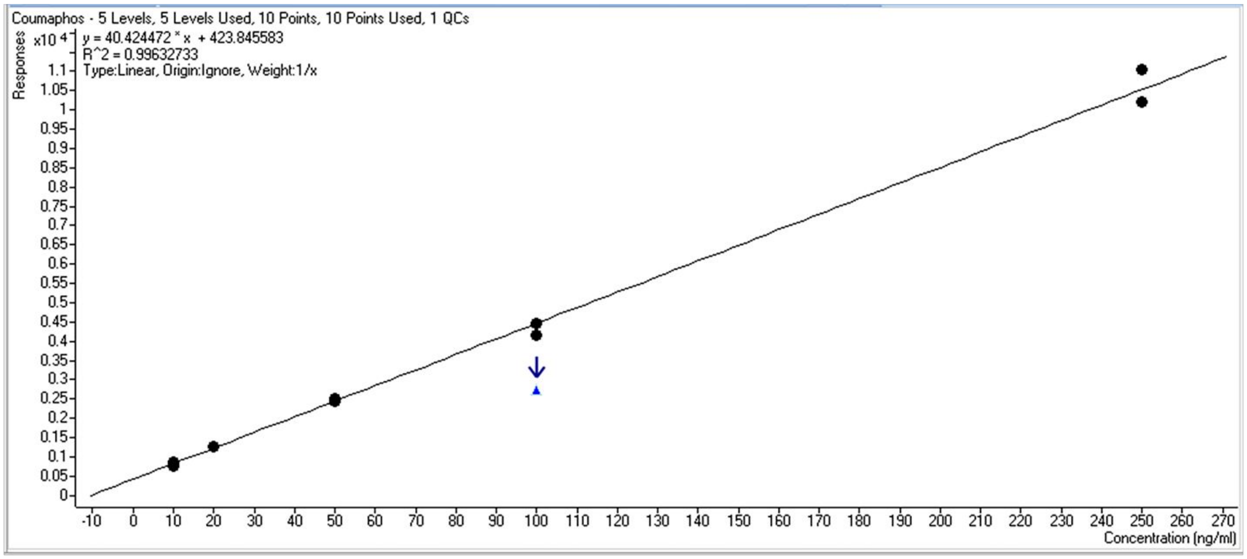


**Figure – S1.** Calibration curve generated for coumaphos. The $R^2$ is considered acceptable as it is higher than 0.950. The GC spike was matrix matched with honey and extracted using the modified NL-method. The illustration was taken from the software Agilent MassHunter and generated using the quantification method.

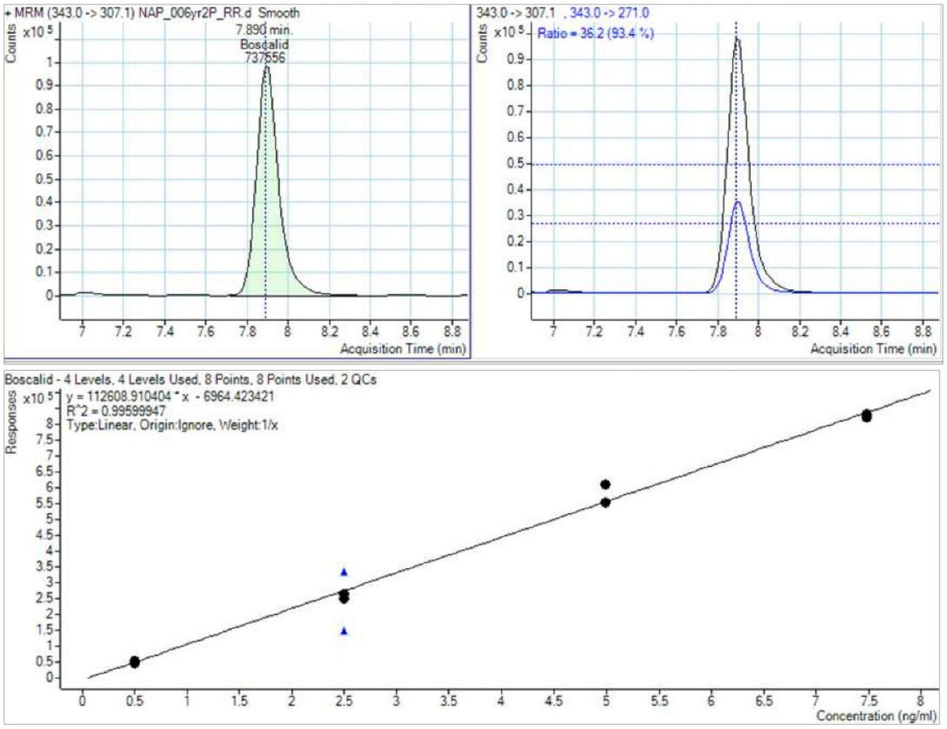


**Figure – S2.** Chromatogram and calibration curve of boscalid detected in a pollen sample through UHPLC-MS analysis in positive electrospray ionisation (ESI) mode. Sample was extracted using the modified NL-method.

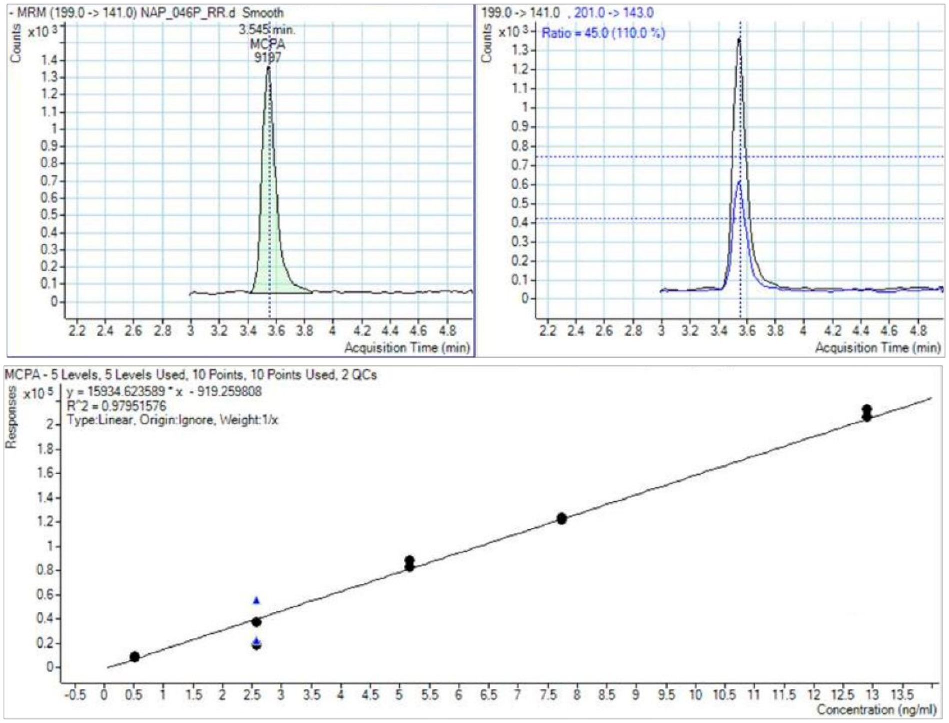


**Figure – S3.** Chromatogram and calibration curve of MCPA detected in a pollen sample through UHPLC-MS analysis in negative ESI mode. The sample was extracted using the modified NL-method.

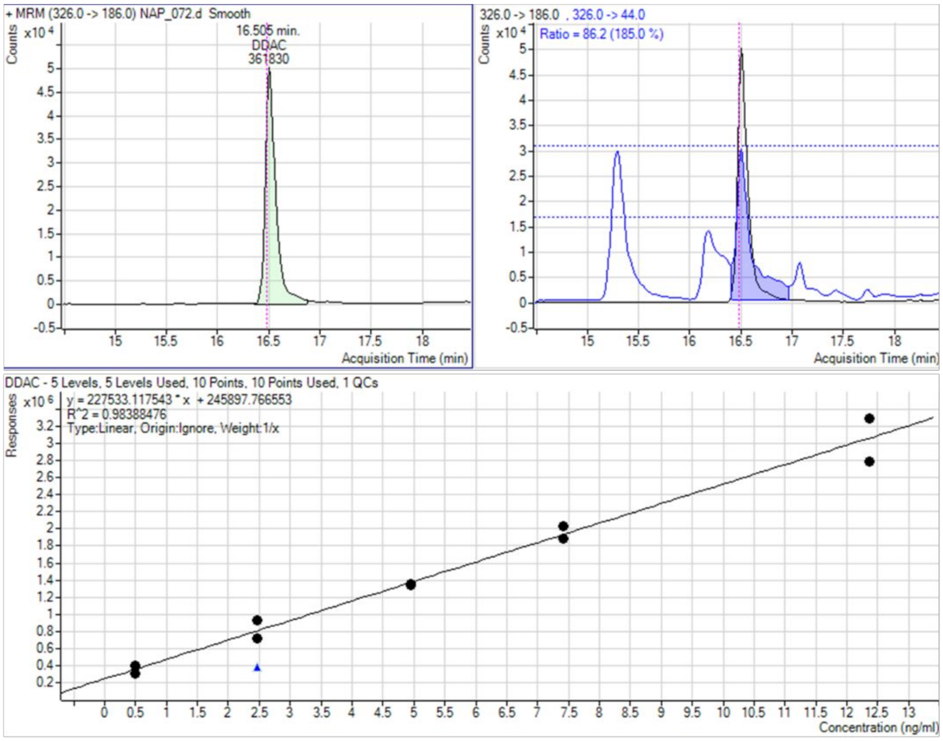


**Figure – S4.** Chromatogram and calibration curve of DDAC detected in honey through UHPLC-MS analysis in positive ESI mode. The honey sample was extracted using the modified NL-method.

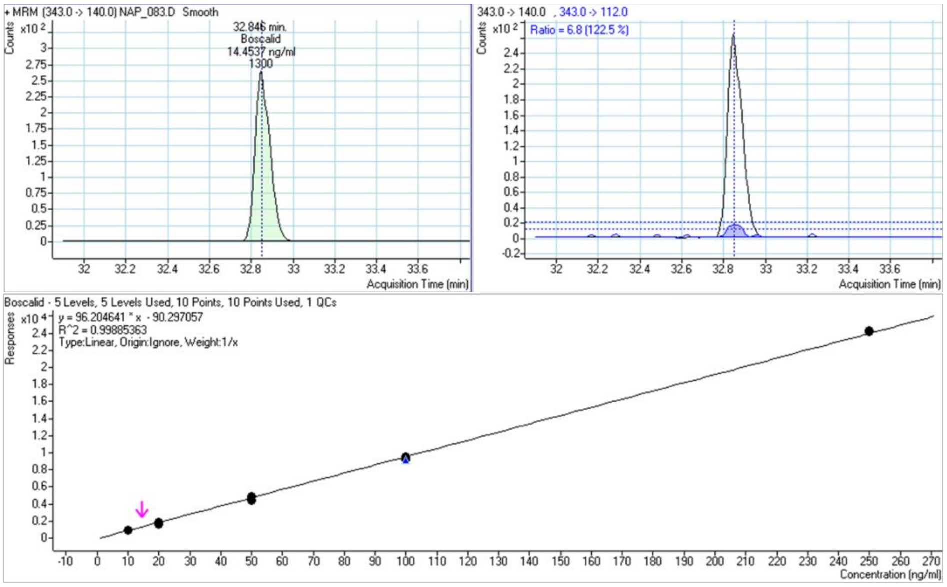


**Figure – S5.** Chromatogram and calibration curve of boscalid detected in a honey sample through GC-MS/MS analysis. The sample was extracted using the modified NL-method.
